# Integrated decision support system for optimizing time and cost trade offs in linear repetitive construction projects

**DOI:** 10.1038/s41598-025-02837-8

**Published:** 2025-06-20

**Authors:** Ahmed Gouda Mohamed, Ali Hassan Ali, Ahmed Adel Abdelhady

**Affiliations:** 1 Construction Engineering and Management Department, Civil Engineering, Faculty of Engineering, The British University in Egypt (BUE), El Sherouk City, 11837 Cairo Egypt; 2 Department of Building and Real Estate, The Hong Kong Polytechnic University, Hong Kong Special Administrative Region of China, Hong Kong, China

**Keywords:** Linear repetitive projects, Time-cost trade-off (TCT), Metaheuristic optimization, Genetic algorithm (GA), Line of balance (LOB), Evolution, Engineering, Mathematics and computing

## Abstract

Linear repetitive construction projects present unique challenges in optimizing both completion time and cost performance. Traditional scheduling techniques often struggle to effectively address these complexities. This paper aims to enhance project optimization by introducing a metaheuristic-based Time-Cost Trade-off (TCT) framework specifically designed for repetitive project environments. Unlike previous studies that focus solely on single-algorithm applications, this research evaluates two metaheuristic optimization strategies—Genetic Algorithm (GA) and Particle Swarm Optimization (PSO)—within a consistent problem setting. The framework employs both algorithms, which are independently assessed for their effectiveness in tackling the Linear Repetitive Project Time-Cost Trade-off (LRPTCT) problem. The methodology utilizes task decomposition alongside the Line of Balance (LOB) scheduling technique, facilitating a more detailed and adaptable planning process. Each sub-task is systematically evaluated to identify the optimal construction method based on cost-time trade-offs, with scheduling constraints integrated into the fitness functions of both GA and PSO. Results from an in-depth case study reveal significant improvements in project efficiency. Specifically, GA achieved approximately a 3.25% reduction in direct costs, a 20% reduction in indirect costs, and a 7% reduction in total construction costs. In comparison, PSO demonstrated slightly superior cost performance, with a 4% reduction in direct costs and comparable reductions in indirect costs, along with a 20% decrease in total project duration. These findings highlight practical gains in resource utilization and scheduling efficiency. This study presents a structured, comparative analysis of GA and PSO within the LOB-based TCT framework, providing a replicable methodology for optimizing schedules in linear repetitive projects. By bridging the gap between traditional scheduling techniques and advanced optimization algorithms, this research contributes valuable insights for enhancing operational efficiency and informed decision-making in construction project management.

## Introduction

The construction industry represents 13% of the global gross domestic product^1,2^. Thus, it plays a highly significant role in economies and helps in improving the standards of living^3-5^. Nevertheless, the scope of this sector is significantly threatened by the challenges that appear in light of the process of urbanization, which increases the pressure on the efficiency of infrastructure construction^6-8^. As stated earlier, there is a pressing need for infrastructure development with commendable emphasis placed on linear repetitive works like bridges, tunnels, and highways, among others, in anticipation of the exploding population globally^9^.

Linear repetitive projects possess distinct characteristics that make them highly suitable for large-scale infrastructure developments. These projects involve sequentially repeated activities across multiple units or sections, ensuring consistency in execution. One of their key attributes is resource continuity, where work crews and equipment transition smoothly between repetitive units, minimizing idle time and improving overall productivity^10^. Additionally, the predictability and standardization of tasks enable more accurate planning, scheduling, and cost estimation, reducing uncertainties compared to non-repetitive projects. The structured nature of these projects also facilitates efficient resource utilization, as labor, materials, and equipment can be allocated systematically to optimize workflow.

The structure of linear repetitive projects offers significant benefits, particularly in enhancing efficiency and cost-effectiveness. The continuity of work improves crew productivity by minimizing disruptions and allowing for better specialization in task execution. Moreover, the Line of Balance (LOB) methodology can be effectively applied to maintain a steady production rate, ensuring that activities across different units progress synchronized^11^. Cost efficiency is another advantage, as repetitive projects benefit from economies of scale, where standardized processes and bulk material procurement lead to substantial savings. Furthermore, the ability to decompose tasks into smaller sub-units provides greater flexibility in project execution, allowing for better control over scheduling and resource allocation. However, despite these advantages, linear repetitive projects require meticulous planning to address potential challenges such as resource bottlenecks and scheduling conflicts. Implementing effective strategies can mitigate these challenges, ensuring the successful execution of linear, repetitive construction projects^11^.

Over the past few decades, the construction industry has faced several issues that make project management a nightmare. Some of the most significant challenges include scope creep, distribution of resources, quality assurance, scheduling, and TCT. For example, scope creep brings in additional costs or durations; ineffective management of the resources may incur more time, and poor quality^12,13^. Managing these competing demands is paramount, especially given that construction projects are usually undertaken within tight schedules and a fixed amount of funds.

The essence of applying the TCT approach is determining the optimum time-cost trade-off of each activity in a project. This strategy aims to reduce the total cost of a project while retaining prescribed timelines for the completion of the project. At the same time, it has to generate schedules that enable tasks to be accomplished at the earliest possible times. This is possible when the beginning of each activity is well determined and the precedences of the tasks and available resources are considered^14^.

Critical path method (CPM) and LOB are conventional scheduling modes commonly used in construction management. However, the accuracy of CPM scheduling conflicts with the method used to schedule repetitive projects that involve repeatedly performing the same basic unit. Still, the main drawback of CPM in this respect is the inability to maintain the continuity of crew work, which is critical for improving the performance rates of repeated processes^15^.

However, as has already been seen, LOB is the best scheduling tool for linear repetitive projects, but it has several drawbacks. LOB sequentially arranges work activities. This might not adequately cover a complex linear work dependent on other linear undertakings. In addition, in LOB, the estimated production rate for each planned activity is assumed to remain constant. Unfortunately, this premise often does not consider actual project environment factors, such as weather conditions or the availability and cost of labor and materials, which can significantly impact project duration^16^. Moreover, the chronological arrangement of activities in LOB may reduce various dependencies and project details embedded within construction projects because dependent tasks are posed linearly. Such distortion leads to improper timing and insufficient coverage of critical and non-significant sub-tasks, which directly endangers project success^17^.

Time and cost control remain crucial in linear repetitive projects since they are usually inversely proportional. Approaches like having more people in the workforce, using non-contemporary building methodologies, or obtaining approval for overtime can shorten the project span and raise costs substantially. However, project managers must factor time and cost together, especially when planning and scheduling, to improve the project performance. Nevertheless, the multiple attribute perspective of the TCT problem can be a drawback as it hinders decision-making. As seen in the segmented decision-making diagram above, many options can be included regarding the time and cost of each activity, and with more possibilities for activities and decisions, the search space size is significantly increased. The combination of parameters in such a manner has been the subject of research in the past few years^18^.

Traditional scheduling methodologies, such as the Critical Path Method (CPM) and Line of Balance (LOB), exhibit limitations when applied to linear, repetitive construction projects that involve complex trade-offs between time, cost, and resource allocation. These conventional techniques often fail to model the dynamic interdependencies among sequential tasks, particularly when resource continuity and crew movement must be optimized. Despite the increasing complexity of construction environments, there remains a notable gap in the literature regarding integrated frameworks that combine Time-Cost Trade-off (TCT) analysis with LOB scheduling principles. Moreover, the potential of advanced metaheuristic algorithms, such as Genetic Algorithms (GA) and Particle Swarm Optimization (PSO), to solve such multi-objective problems remains underutilized. This shortfall highlights the need for a more robust and adaptive dual-objective optimization framework that addresses project duration and cost efficiency in repetitive construction workflows.

The primary contribution of this research lies in developing an integrated decision-support system that optimizes the TCT in Linear Repetitive Projects (LRPs) by combining GA and PSO with the LOB methodology. Unlike previous studies that either focus solely on conventional scheduling techniques like the CPM and LOB or optimize TCT without considering the unique characteristics of repetitive projects, this research bridges a critical gap by introducing a dual-optimization framework tailored explicitly for LRPs. A key novelty of this study is the decomposition of repetitive tasks into sub-tasks, which allows for a more granular analysis of scheduling constraints, resource allocation, and cost optimization. This approach has not been extensively explored in existing literature. The proposed framework successfully integrates metaheuristic optimization techniques with repetitive construction scheduling, enabling more precise trade-off analysis between time and cost while ensuring continuous workflow and efficient crew utilization. The comparative evaluation of GA and PSO further enhances the study’s impact by offering insights into their respective strengths, with GA excelling in exploring diverse solution spaces, while PSO demonstrates faster convergence and superior cost efficiency. By incorporating LOB-based decomposition and multi-objective optimization, this research presents a scalable and practical tool for improving project scheduling and cost management in linear repetitive construction projects, marking a significant advancement over existing approaches.

Existing studies on TCT optimization in LRPs have primarily focused on conventional scheduling methods such as the CPM and LOB or have applied metaheuristic optimization techniques without explicitly considering the unique characteristics of repetitive construction projects. While CPM is widely used, it struggles to maintain crew continuity, a critical factor in repetitive construction, leading to inefficiencies. Although LOB provides a structured approach to scheduling, previous studies have oversimplified repetitive tasks as uniform activities, neglecting sub-task decomposition and its impact on time-cost efficiency. Furthermore, most prior optimization models have focused on either GA or PSO separately, without a direct comparative analysis of their performance in LRPs. This research addresses these gaps by developing a novel integrated decision-support framework that combines GA and PSO with LOB scheduling, allowing for a more granular and dynamic optimization process. Unlike past studies, the proposed model decomposes repetitive tasks into sub-tasks, capturing variations in production rates, resource allocation, and scheduling flexibility, leading to more precise time-cost trade-offs. Additionally, this study provides a comprehensive performance comparison between GA and PSO, demonstrating their respective strengths in exploring solution spaces and achieving cost-efficient scheduling. By bridging the gap between metaheuristic optimization and LOB-based scheduling, this research introduces a scalable and practical approach that significantly improves project efficiency, cost savings, and execution strategies for linear, repetitive construction projects.

The significance of this research extends well beyond its theoretical contributions. It redefines the integration of TCT and LOB, presenting a transformative decision-support tool that empowers project managers to optimize linear repetitive projects with unprecedented precision. This framework opens new avenues for addressing complex scheduling and cost management challenges, offering a scalable, flexible, and impactful solution that can be adapted across diverse project scenarios. By advancing the state of the art in construction project management, this study lays the groundwork for future innovations in multi-objective optimization, reinforcing its uniqueness and practical value to the field.

## Literature review

Construction projects can be broadly classified into two main categories: linear and non-linear. Linear projects are executed in a sequential manner and are characterized by a clear, straightforward progression. Examples of such projects include transport corridors, specifically pipelines, highways, and railroads, where the completion of each segment is essential for the initiation of the next. This method is conducive to efficiency and predictability, facilitating effective resource allocation and scheduling^19^. In contrast, non-linear projects encompass constructions that are not restricted to a single sequence. These projects often involve multiple structures, which may exhibit diverse designs and complexities, such as residential developments featuring numerous houses or multiple high-rise buildings. Management of non-linear projects requires a more flexible approach due to the simultaneous execution of various tasks and construction activities^15^.

Currently, repetitive activities play a vital role in nearly all construction processes, particularly in large-scale operations. Many of these projects exhibit cyclical patterns of activity, where the continuous use of resources significantly enhances both efficiency and effectiveness^20^. This continuity is crucial, as repetitive work involves performing tasks multiple times. By adopting this approach, projects can achieve substantial cost and time savings, primarily due to the establishment of a consistent, skilled labor crew that remains engaged throughout the project duration^21^. However, implementing this strategy also presents challenges. The repetitive nature of the work can lead to diminished productivity over time, as crews may experience fatigue or monotony. Additionally, transitions between units can prolong the overall timeline and increase expenses, as crews must repeatedly execute the same tasks^22^. Therefore, balancing the benefits of continuity with the potential drawbacks of reduced productivity is crucial for optimizing project outcomes. Consequently, it is vital to develop an efficient plan for crew work in repetitive projects to achieve optimal performance in terms of cost and duration^23^.

This balance is where time-cost trade-off problems (TCTP) become particularly relevant. TCTP focuses on optimizing the trade-offs between project duration and associated costs, making it essential for both linear and non-linear projects^20^. In construction, effectively managing TCTP can lead to improved resource utilization and enhanced scheduling efficiency. Recently, a substantial body of research has emerged that addresses time-cost trade-off problems. These studies can be broadly categorized into two distinct groups: those that explore general construction projects and those that specifically examine repetitive construction projects. This categorization clarifies the different approaches and considerations involved in managing time and cost across various types of construction projects.

### General construction projects

This section will focus on the application of TCTP across various construction contexts, emphasizing optimization models, uncertainty management, and algorithm performance. General construction projects encompass a wide range of types, including residential, commercial, industrial, and infrastructure initiatives. Each project typically features unique specifications, designs, and requirements, which can lead to variability in both time and cost. Scheduling in these projects often involves diverse tasks and dependencies, necessitating tailored approaches to effectively manage timelines. This section will highlight general optimization techniques that address the uncertainties and risks associated with individual projects, ensuring a more robust and efficient project management strategy.

For instance,^24^ address the critical TCTP in general construction projects, emphasizing the significant impact of uncertainties on project management outcomes. Their study introduces a multiobjective robust optimization model that incorporates interval uncertainties in both time and cost parameters. This innovative approach enhances the understanding of tradeoffs by accounting for the variability inherent in project execution. The authors utilize a modified nondominated sorting genetic algorithm-II (NSGA-II) to derive robust Pareto solutions, providing decision-makers with a set of viable construction alternatives based on varying acceptable risk levels. This methodological advancement not only aids in effective decision-making but also offers a practical tool for managing uncertainties in construction projects. However, the study has limitations, particularly regarding the assumptions about the nature of uncertainties and the specific context of the engineering example used. Further empirical validation across diverse project scenarios is essential to enhance the applicability of their findings. Additionally, exploring the implications of resource constraints within their robust optimization framework could yield further insights. Overall, this study contributes a valuable framework for robust optimization in construction management, laying the groundwork for future research in this area.

Additionally,^25^ investigate the critical relationship between time and cost in construction project management, addressing the complexities of meeting project objectives within budget constraints. They utilize a hybrid model that combines the CPM with a heuristic approach known as the cost-loop method to analyze the trade-offs involved in project execution. The study effectively illustrates the importance of balancing time and cost through regression analysis, which establishes a clear relationship between crash times and costs, ultimately leading to an optimized linear programming model. The results reveal a minimum total cost of $60,937 for a project duration of approximately 130 days, demonstrating the practicality of their approach in real-world scenarios. However, the study’s exclusive focus on deterministic values limits its applicability in environments characterized by uncertainty and variability, which are common in construction projects. This constraint also affects the external validity of the study’s outcomes. To address uncertainties in project parameters, future research could benefit from integrating probabilistic models or fuzzy logic. Overall, this study provides valuable insights into time-cost trade-off analysis, offering a robust framework for practitioners to enhance decision-making in construction management. Its findings highlight the necessity for further exploration of uncertainty in future studies to improve predictive accuracy.

^26^ introduce a novel approach to solving Time-Cost-Environmental impact Trade-Off (TCET) optimization problems in construction projects by utilizing a modified Teaching-Learning-Based Optimization (TLBO) algorithm enhanced with a Golden Ratio-based Oppositional Learning (GROL) strategy. This study addresses the limitations of existing TLBO variants, particularly their susceptibility to local optima and slow convergence rates in complex optimization scenarios. By incorporating the golden ratio into the opposition-based learning framework, the authors significantly enhance the algorithm’s exploration capabilities, which is essential for effectively navigating the multi-dimensional solution space of TCET problems. The empirical results reveal a 5% improvement in accuracy over competitive algorithms, while also substantially reducing the number of scheduling calculations required. However, the proposed Golden Ratio Oppositional TLBO (GROTLBO) primarily demonstrates its effectiveness through benchmark problems, which may not fully encapsulate the complexities of real-world construction projects. Additionally, one important issue pertains to the computational time, which is influenced by the algorithm’s search space and the characteristics of the optimization problem. Future studies should evaluate the algorithm’s performance in diverse, large-scale scenarios and incorporate uncertainty analysis to further validate its robustness. Overall, this research offers valuable insights into multi-objective optimization in construction management, emphasizing the importance of environmentally conscious decision-making while balancing time and cost objectives.

Similarly,^27^ present a significant advancement in the field of construction project management through their development of the Modified Dynamic Opposition Learning-assisted Teaching-Learning-Based Optimization (MDOLTLBO) algorithm. This study addresses the complexities of TCTP in construction projects by incorporating a dynamic opposition learning strategy, which enhances population initialization and improves convergence speed. The authors convincingly argue that traditional optimization methods often struggle with local optima and slow convergence, particularly in large-scale construction projects. By utilizing modified dynamic opposite points, the MDOLTLBO algorithm increases solution diversity and effectively narrows the search space, thereby facilitating more efficient optimization processes. The empirical results demonstrate the algorithm’s effectiveness across various case studies, including projects with up to 290 activities, showcasing its ability to deliver high-quality, non-dominated solutions. However, while the findings are promising, the study could benefit from a broader examination of real-world applications and the integration of uncertainty analysis to enhance robustness. Additionally, the performance of this heuristic is influenced by the specifics of construction projects and problem instances. Although MDOLTLBO improves convergence and solution quality, it may still become trapped in local optima within large-scale, high-dimensional search spaces. Overall, this research contributes valuable insights into multi-objective optimization in construction, highlighting the importance of balancing time, cost, and resource utilization while addressing the challenges of generalized precedence relations. Future studies should explore the algorithm’s performance in more complex construction scenarios to further validate its applicability.

### Repetitive construction projects

This section will explore the unique challenges and solutions related to TCTP in repetitive construction scenarios, focusing on specialized algorithms and scheduling techniques. Repetitive construction projects typically involve the repetition of similar tasks or modules, as seen in residential developments and large-scale infrastructure projects. These projects often use standardized designs and construction methods, which allow for more predictable scheduling and budgeting. Scheduling in these projects is often more straightforward than in general construction projects due to the repetitive tasks, enabling the use of specific techniques like LOB. This section will emphasize the specialized algorithms that consider the unique characteristics of repetitive tasks, including sequencing and resource allocation across multiple units.

For example,^28^ tackle the discrete time-cost trade-off problem (DTCTP) in repetitive construction projects by introducing a genetic algorithm-based method that incorporates soft logic to accommodate variable work sequences. This approach acknowledges that traditional optimization techniques often simplify work sequences, potentially leading to suboptimal scheduling outcomes. By formulating a mathematical model that considers different work sequences, the authors offer a more flexible framework for scheduling activities across multiple units, particularly relevant in projects such as highways and housing developments. Their targeted genetic algorithm focuses on encoding activity modes and work sequences while determining suitable start times through linear programming, enhancing computational efficiency. Results from two case studies demonstrate the effectiveness of this method in minimizing costs and meeting deadlines, supporting the assertion that soft logic can significantly improve project scheduling. When compared to a previous scheduling solution that utilized only hard logic, their findings indicate greater competitiveness in solutions using soft logic—one of which reduced actual project time by eleven days and saved $25,283 through the application of different work sequences. However, while the findings are promising, the study primarily relies on specific project examples, which may limit the generalizability of the results. Additionally, the presented mixed-integer nonlinear programming model can be computationally intensive and sensitive to project scale. Although the genetic algorithm is generally robust in generating high-quality solutions, its performance can vary based on parameters such as population size and mutation rates. Future research could expand on this work by incorporating larger datasets and more complex project scenarios to further validate the proposed method’s applicability across diverse construction contexts. Overall, this study provides valuable insights into optimizing time-cost trade-offs in construction management.

^29^ explore the mixed-integer linear programming (MILP) approach for scheduling repetitive construction projects, focusing on time-cost trade-offs and the utilization of multiple crews. This study addresses a significant gap in the existing literature, which often overlooks the complexities involved in scheduling repetitive projects where activities can be performed simultaneously by different crews. By formulating a rigorous mathematical model that incorporates fixed logic and multiple crew assignments, the authors present a systematic framework for minimizing total project costs while adhering to deadlines. Their computational experiments demonstrate the effectiveness of the proposed exact model for medium-sized problems, as well as an approximate model that provides efficient solutions for larger instances. The findings indicate that the exact model can effectively manage the complexities of repetitive project scheduling, while the approximate model offers a practical solution for larger-scale projects. Simulations reveal that the approximate model maintains a maximum deviation of less than 1% and outperforms the exact model for larger problem sizes. However, the study’s assumptions, particularly regarding the use of a single execution mode for activities, may limit its applicability in more diverse project scenarios. Future research could enhance this model by incorporating variable execution modes and exploring its performance in real-world applications. Overall, this study contributes valuable insights into optimizing project scheduling, emphasizing the importance of considering multiple operational factors in construction management.

^30^ present an innovative optimization model for scheduling repetitive construction projects, uniquely focusing on minimizing interruption costs in addition to project duration and work interruptions. This study addresses a critical limitation in existing models, which often fail to account for the financial impact of crew downtime or relocation caused by work interruptions. By employing a two-step procedure, the model first generates schedules that minimize project duration and work interruptions, and then optimizes these schedules to minimize interruption costs using a single-objective optimization approach. The model’s computations are structured around four modules: optimization, initial scheduling, intermediate scheduling, and interruption cost calculation, providing a comprehensive framework for schedule optimization. The application of genetic algorithms allows the model to effectively handle the non-linear relationships inherent in construction scheduling, while considering factors such as crew movement costs, idle crew costs, and minimum work durations. The results demonstrate the model’s capability to generate schedules with significantly lower interruption costs compared to existing models that primarily focus on duration and work continuity. However, the model’s reliance on serial relationships among project activities may limit its applicability in projects with more complex dependencies. Furthermore, pre-restricting activities to a single crew formulation and maintaining serial relationships may reduce its scope in complex projects. Future research could expand the model to accommodate non-serial activities and explore its performance in real-world construction scenarios. Overall, this study contributes valuable insights into optimizing repetitive construction project scheduling, highlighting the importance of considering interruption costs for improved project efficiency and cost management.

^31^ introduce a multi-objective mixed-integer linear programming model to address the repetitive project scheduling problem, considering work continuity constraints and soft logic. This research builds upon existing literature by allowing the logic relation between units to be changed arbitrarily and incorporating multi-crew execution, thereby enhancing flexibility and practicality. By formulating a mathematical model that optimizes the tradeoff among project duration, work interruptions, and total project cost, the authors provide a comprehensive framework for generating Pareto-optimal solutions using a customized efficient version of the ε-constraint algorithm. The model’s validation through case studies demonstrates the influence of soft logic on project duration and total cost, especially in projects with multiple crews. This highlights the model’s effectiveness in minimizing these objectives, particularly for projects with a high percentage of non-typical activities. However, the study’s limitation lies in neglecting the learning-forgetting phenomenon, which could impact activity durations and crew productivity. Future research could enhance the model by incorporating these effects and exploring its applicability in diverse project scenarios. Overall, this study contributes a novel soft logic-based mathematical programming model for repetitive project scheduling, offering a more comprehensive approach to optimizing conflicting objectives simultaneously and providing valuable guidance for practitioners.

^15^ present an optimization model for time-cost trade-offs in recurring projects, considering interruption, buffer, and schedule acceleration. This study addresses a limitation in previous models that often focus on a single aspect of optimization, such as interruption or buffering. By developing a model with modules for scheduling and interruption, scheduling and buffering, and schedule acceleration, the authors provide a more comprehensive approach. The unit-based acceleration algorithm and queuing criteria, which incorporate both cost slope and contractor’s judgment, are notable contributions. The model’s flexibility, allowing users to choose interruption percentages, overtime factors, and relative weights, enhances its practical applicability. The authors automate the model using C# programming language, making it suitable for large-size projects. However, the study’s reliance on a single-objective optimization approach, addressing either cost or duration but not both concurrently, presents a potential limitation. Additionally, its dependence on certain quantifiable factors may not fully capture real-world complexities, potentially leading to gaps in the development of a full range of solution possibilities. Also, depending on the input data provided, the model functionality is rather sensitive and may change from one project to another. Future research could explore multi-objective optimization techniques to provide a more comprehensive set of trade-off solutions. Overall, this study contributes a valuable tool for optimizing recurring project schedules by integrating multiple factors and offering a flexible framework for decision-making.

^20^ investigate the multi-objective TCTP in construction scheduling using Rao-1 and Rao-2 algorithms integrated with a non-dominant sorting (NDS) method. This study addresses the challenge of selecting optimal time-cost options for activities, a combinatorial problem that increases in complexity with the number of activities and options. By integrating NDS into the Rao series, the authors aim to provide decision-makers with multiple Pareto-optimal solutions, offering a range of choices based on their experience and knowledge. The numerical results demonstrate that the NDS-Rao-2 algorithm outperforms algorithms like PSO, Ant Colony Optimization (ACO), and GA, finding better results with a significant number of function evaluations. The study highlights the ability of the NDS-based Rao-2 algorithm to not only find optimal/near-optimal results but also suggest multiple Pareto solutions, enhancing its practical applicability. However, the study concentrates on small and medium-scale problems. Moreover, it primarily focuses on discrete optimization scenarios, which may not suit all construction projects, particularly those involving continuous variables. Additionally, the performance of the Rao algorithms is influenced by the quality of the initial population and parameter settings, which can affect convergence rates. Future research could expand the model by testing its performance on larger and more diverse datasets. Overall, this study contributes a valuable alternative for solving time-cost trade-off problems, showcasing the effectiveness of the multi-objective Rao-2 algorithm in providing a range of Pareto-optimal solutions for construction management decision-making.

^32^ introduce a novel multiple objective whale optimization (MOWO) algorithm for the time-cost-quality trade-off (TCQT) problem in non-unit repetitive projects (NRP). This study addresses the limitations of existing methods that assume uniform units and neglect the sequence of activities in each unit. By developing a free-parameter MOWO and a scheduling subsystem, the authors aim to optimize project duration, cost, and quality while considering activity dependencies, multiple crew assignments, and soft logic in units. The construction case study demonstrates that MOWO achieves better optimization outcomes compared to other multiple objective evolutionary algorithms. The algorithm’s ability to minimize project time and cost while maximizing quality through optimal crew assignment and activity sequencing is a significant contribution. However, the study’s reliance on a single case study and the lack of a detailed sensitivity analysis may limit the generalizability of the findings. Future research could expand the model by testing it on more diverse datasets. Overall, this research contributes a valuable tool for solving the TCQT problem in NRP, showcasing the effectiveness of the MOWO algorithm in construction management.

Table 1 presents a comprehensive overview of several research papers, meticulously summarizing their core objectives. Each entry delves into the nuanced details of the study, elucidating the trade-offs inherent in the research design, the specific categories of projects under investigation, the particular problems and challenges addressed by the researchers, and the methodologies and techniques employed to tackle these issues and achieve the research goals. This detailed summary allows for a quick and efficient understanding of the key aspects of each research paper, facilitating comparative analysis and knowledge synthesis.

**Table 1:** Summary of previous research.

Number	Reference	Tradeoff	Type of projects	Problem	Methods
1	^24^	Time, Cost	General construction projects	Uncertainty	NSGA-II Optimization
2	^25^	Time, Cost	General construction projects	Deterministic values	Hybrid CPM with Heuristic Cost-Loop
3	^26^	Time, Cost, Environmental	General construction projects	Multi-objective, Dynamic	GROTLBO Algorithm
4	^27^	Time, Cost	General construction projects	Multi-objective, Dynamic	MDOLTLBO Algorithm
5	^28^	Time, Cost	Repetitive projects	Repetitive, Soft logic	Targeted genetic algorithm
6	^29^	Time, Cost	Repetitive projects	Repetitive, Fixed logic	MILP approach (Exact and Approximate)
7	^30^	Time, Cost, Interruption	Repetitive projects	Repetitive	Genetic algorithm model
8	^31^	Project Completion Time, Total Interruption Time, Total Project Cost	Repetitive projects	Repetitive, Soft logic	Multi-objective MILP Model
9	^15^	Time, Cost	Repetitive projects	Recurring, Interruptions, Buffers	Integrated scheduling optimization Model
10	^20^	Time, Cost	Repetitive projects	Multi-objective Optimization	Non-dominated sorting with Rao algorithms
11	^32^	Time, Cost, Quality	Repetitive projects	Non-unit repetitive Projects with soft logic, Optimal crew assignment and activity sequencing	MOWO

In summary, while existing literature provides valuable insights into cost optimization and scheduling in construction projects, a significant gap remains. Specifically, there is a lack of documented methodologies that effectively integrate time-cost trade-offs (TCT) with the visual planning capabilities of Line of Balance (LOB) techniques, all within the adaptive framework of the GA and PSO. This gap is particularly noteworthy, as combining these methods could offer a more holistic perspective on project optimization. Such an integrated approach has the potential to substantially enhance both the efficiency and effectiveness of construction projects, especially in repetitive settings where similar tasks and workflows are common. To address this critical research gap and advance the field, this study aims to develop an innovative and integrated decision-support system designed to leverage the strengths of TCT analysis and LOB planning, effectively merging them within a well-structured GA and PSO framework. This integrated approach seeks to equip decision-makers, including project managers, engineers, and other key stakeholders, with a robust and versatile toolkit. By enhancing capabilities for optimizing resource allocation, managing project timelines, and mitigating potential risks, this research ultimately aims to improve project outcomes characterized by reduced costs, minimized delays, and a higher likelihood of overall success.

## The mathematical formulation of time cost trade-off

The optimization problem aims to minimize project duration and total cost by selecting construction methods for each repetitive sub-task. Each technique has predefined duration and cost values, represented by the decision variable xi∈{1,2,3,4. The first objective minimizes the total project duration based on task sequencing; the second minimizes the total cost, combining direct costs and time-based indirect costs. Constraints enforce logical precedence, method selection limits, and planned time and cost adherence. Assumptions include task decomposition into equal sub-units, fixed dependencies, and deterministic input values. This formulation underpins the GA and PSO algorithms applied in the next section.

Multi-objective optimization is pivotal to addressing the time-cost trade-off problem in project scheduling. The goal is to minimize the total project duration and the associated costs by selecting the most appropriate construction method (or resource option) for each activity. To accomplish this, time calculation becomes fundamental, as it directly influences the project delivery schedule and the accumulation of indirect costs. The time scheduling component is modeled through a set of core scheduling equations — Eq. (1) to (3) — that determine each activity’s earliest start and finish times and ultimately the project’s completion time. These are fundamental in identifying the critical path, defining the minimum project duration.

Equation 1 computes the Earliest Start (ES) of activity j as the maximum of the Earliest Finish (EF) times of all its immediate predecessors i (i.e., the activities in the set pj). In Eq. 2, the *Earliest Finish (EF)* of an activity I is determined by adding its duration ti(m)corresponding to option m, to its *Earliest Start (ES)*. This allows flexibility in choosing among multiple methods or resources (indexed by m) for each task, influencing time and cost. The total project duration T (Eq. 3) is obtained from the EF of the final dummy activity *n* + 1, which signifies the project’s end. This duration is crucial for calculating the indirect costs and for assessing the overall efficiency of the project schedule.

1 $$ES_{j} = {\text{ }}\max _{{i \in pj}} \left\{ {EF_{i} } \right\}j{\text{ }} = {\text{ }}1, \cdots ,{\text{ }}n + 1$$

2 $$EF_{i} = {\text{ }}ES_{i} + t_{i} ^{{(m)}} i{\text{ }} = {\text{ }}0, \cdots ,{\text{ }}n + 1$$

3 $${\text{T }} = {\text{ EF}}_{{{\text{n}} + {\text{1}}}}$$

The Equations portrayed above involve various parameters, incorporating the total project duration, denoted as T, the activities with which j has a precedence relation depicted by pj, and the activity’s early start and early finish, signified as ESj and EFi, respectively. Additionally, the activity m duration is rendered by ti(m). The primary objective of these equations is to determine the project’s completion time by identifying the project’s longest path. Hence, the aggregate of each activity’s direct costs and the entire duration of the project multiplied by the indirect cost equals the total cost of the project, as explicitly rendered in Eq. (4) to (6). In this regard, DC depicts the direct cost, C represents the entire project’s cost, IC resembles the indirect cost, ICR represents the whole project’s indirect costs, and T represents the entire project’s duration.

4 $$C = \mathop \sum \limits_{{i = 0}}^{{n + 1~}} dci^{ \wedge } \left( m \right)xi^{ \wedge } \left( m \right)$$

5 $${\text{IC }} = {\text{ T}} \times {\text{ICR}}$$

6 $${\text{C }} = {\text{ DC}} + {\text{IC}}$$

## Research methodology

**Fig. 1:**
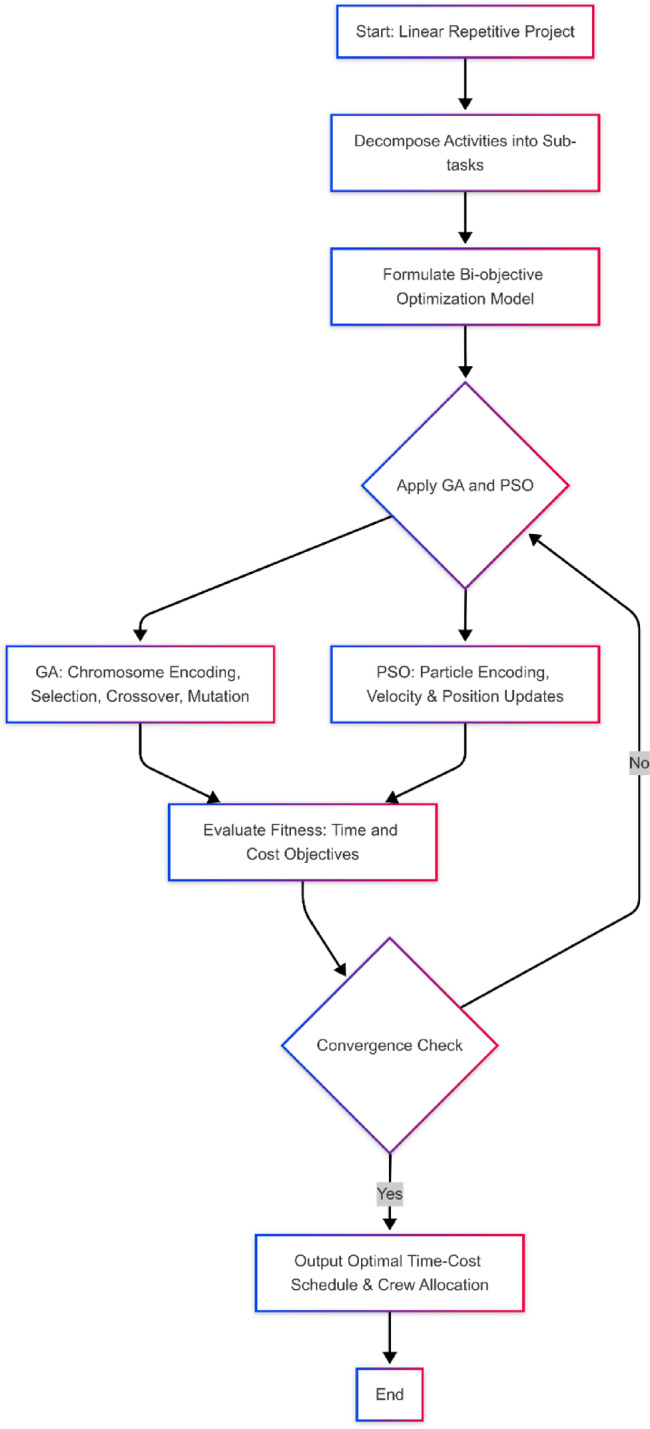
Proposed research methodology.

This study presents a hybrid framework integrating the LOB method with GA and PSO to optimize time-cost trade-offs in linear repetitive projects (see Fig. 1). The process begins by decomposing repetitive activities into sub-tasks based on complexity, resource constraints, production rates, and logical dependencies. This enables flexible scheduling and dynamic crew allocation while preserving workflow continuity.

The problem is formulated as a bi-objective optimization model to minimize total project duration and construction cost. Decision variables represent the selected construction method for each sub-task, subject to constraints on precedence, budget, time, and feasibility. GA encodes solutions as chromosomes and uses selection, crossover, and mutation to evolve toward optimal schedules. PSO represents solutions as particles and updates them iteratively based on personal and global best positions. Both algorithms evaluate fitness using the same objective functions and run until convergence. This integrated approach supports efficient, constraint-aware scheduling under realistic linear construction conditions.

### The devised methodology for scheduling the linear repetitive project

**Fig. 2:**
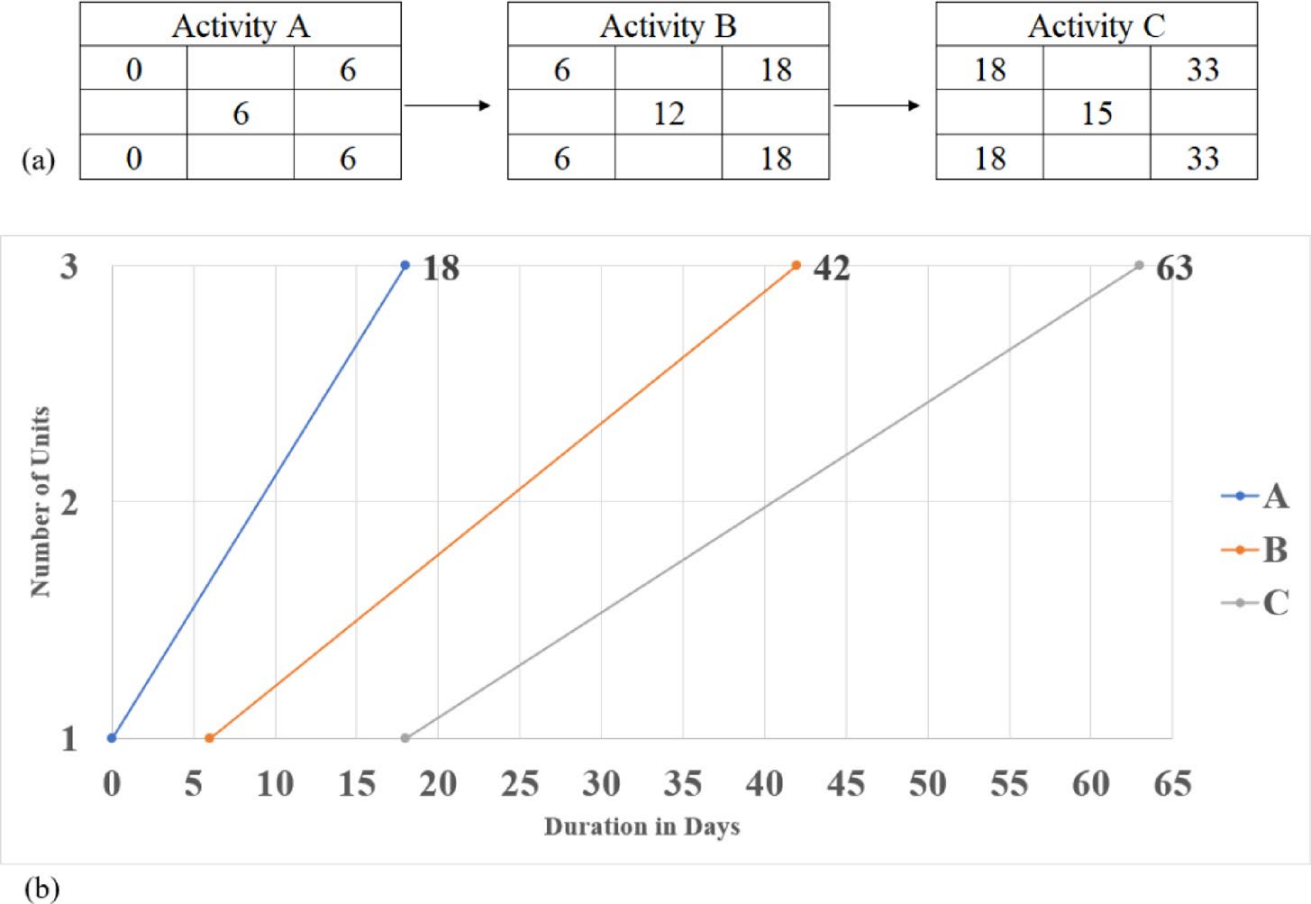
Conventional scheduling using the LOB method; (**a**) A Critical Path Method (CPM) for one unit depicting the criticality of the entire tasks in the unit; (**b**) Exhibiting tasks A, B, and C using a LOB diagram.

Principally, linear construction projects that pursue a linear path routinely encounter hindrances, dictating the incorporation of non-critical or repetitive tasks for remedying resource limitations. Routinely, the LOB scheduling method presupposes that repetitive tasks have a steady production rate, remaining constant across the project duration, irrespective of the desired unit number (See Fig. 2). As reported in Fig. 2a, the completion time of one unit is 33 days; on the other hand, the completion time of the three repetitive units equals 63 days, as rendered in Fig. 2b.

Additionally, LOB scheduling reduces the propensity for repeated tasks to be partially critical because each repeated task is enacted in a single, undivided pattern with a predetermined deterministic duration. This presumption could challenge LOB scheduling to affirm that the same crew constitutes each task linearly, rendering it impractical to disseminate resources across the entire project. This restriction is particularly problematic, as repetitive activities may encompass multiple sub-tasks with varying production rates and entail distinctive crew dissemination. As such, the production rate of each sub-task is imperative for tailoring to optimize resource allocation. Contrary to the conventional method of appraising construction tasks in LOB scheduling, repetitive tasks will be exhibited as a series of distinct sub-tasks interconnected to render the overall duration of the task.

**Fig. 3:**
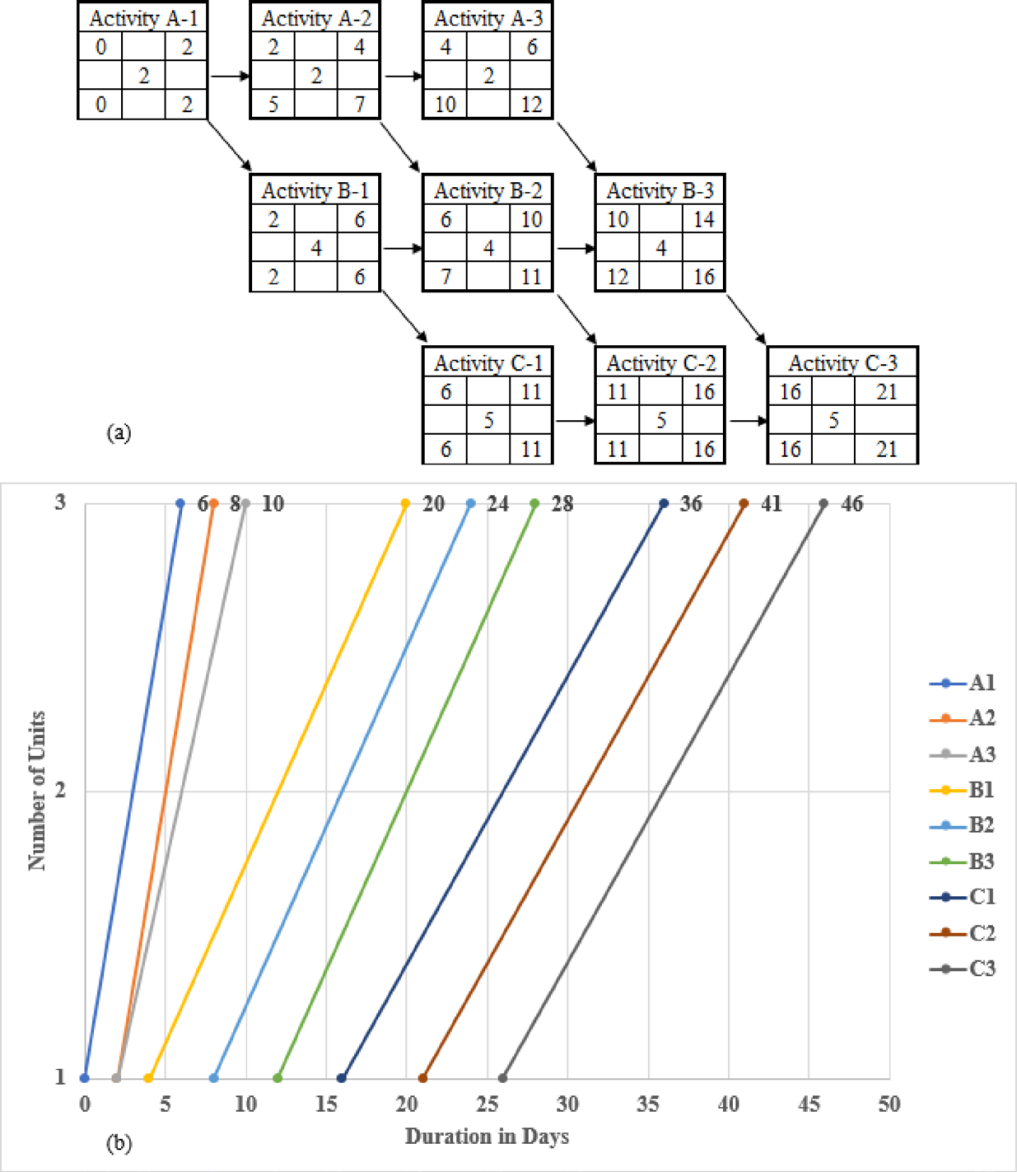
The proposed approach for rendering repetitive tasks: (**a**) Illustrating a CPM for one unit after splitting tasks into sub-tasks; (**b**) portraying tasks A-1, A-2, and A-3 via a LOB diagram after splitting task A into partitioned sub-activities.

This approach depicts any logical connection between the decomposed tasks, adopting solely the finish-to-start link. By decomposing tasks in the LOB diagram, more methodical schedules are engendered and portray critical and non-critical sub-tasks unerringly. Correspondingly, this technique acknowledges the aleatory conditions of construction projects. Even though decomposing tasks may provoke more tasks in LOB scheduling, it can instigate a more profound schedule when resource limitations are present, as shown in Fig. 3. As attested in Fig. 3a, the completion time of one unit is 21 days; contrastingly, the completion time of the three repetitive units equals 46 days, as rendered in Fig. 3b.

The sub-task division is based on four key criteria to ensure consistency and replicability. Activity complexity guides the breakdown of tasks with multiple work phases or varying conditions. Resource constraints influence division when shared crews or specialized equipment are involved. Production rates determine segmentation to align with efficiency and output requirements. Logical dependencies ensure that sub-tasks maintain proper sequencing and constructability. These criteria enhance the adaptability and practicality of the proposed framework in real-world projects.

The proposed LOB-based scheduling framework does not assume a fixed single crew per activity but instead utilizes task decomposition to enable dynamic crew allocation. The model optimizes crew assignments based on cost-time trade-offs rather than enforcing a constant production rate by breaking down repetitive activities into smaller sub-tasks. Integrating GA and PSO ensures that multiple crews can be assigned where beneficial, improving scheduling flexibility and resource utilization. Unlike traditional LOB, which may assume uniform production rates, our framework adapts crew assignments dynamically, balancing efficiency and cost. This clarification distinguishes our approach from the Linear Scheduling Method (LSM) while preserving LOB’s advantage of flexible multi-crew scheduling. The manuscript has been updated to reflect this distinction.

### Genetic algorithm approach for LRPTCT

GA portrays a metaheuristic optimization algorithm stimulated via natural selection and genetics. It is a search technique adopted to retrieve the best resolution for a particular problem by mimicking the natural determination process and evolution^33^. Genetic algorithms operate on a population of prospective resolutions encoded as chromosomes or strings of information. The population endures a series of genetic operations, including selection, crossover, and mutation, to generate a new population of potentially better solutions. The selection process incorporates opting for the fittest individuals from the current population based on their fitness values, exhibiting how thoroughly they resolve the problem. These selected individuals are then employed to generate new solutions through crossover, which involves combining parts of two or more parent solutions to create new offspring^34^.

Another genetic procedure called mutation imparts random alterations to the offspring solutions, incentivizing the exploration of new areas of the solution space. This process empowers a deeper investigation of the solution space and precludes the algorithm from being stuck in local optima. The latest population of solutions is then appraised for fitness, and the process is repeated until a termination criterion is met, such as reaching a maximum number of generations or achieving a desired level of fitness. After the genetic algorithm process, the population member with the highest level of performance is touted as the optimal solution to the optimization^34^.

#### Encoding the chromosome

**Fig. 4:**
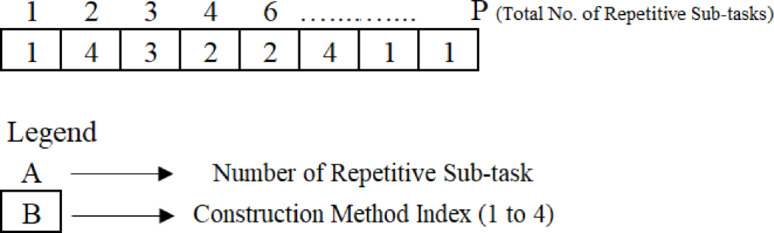
The configuration of chromosomes in the GA problem.

In GA, a solution to an optimization problem is embodied as a “chromosome,” a data structure that encodes a batch of parameters denoting the solution. In the context of time cost trade-off optimization, a chromosome structure was encoded using different configurations of defined construction methods coupled with correlated duration and direct cost. A chromosome structure was encoded by a sequencing chain of elements, with each component portraying a linear repetitive sub-task and including an index indicating its defined construction method. This sequence renders a single project solution, constituting methods to construct the repetitive sub-task, as rendered in Fig. 4.

**Fig. 5:**
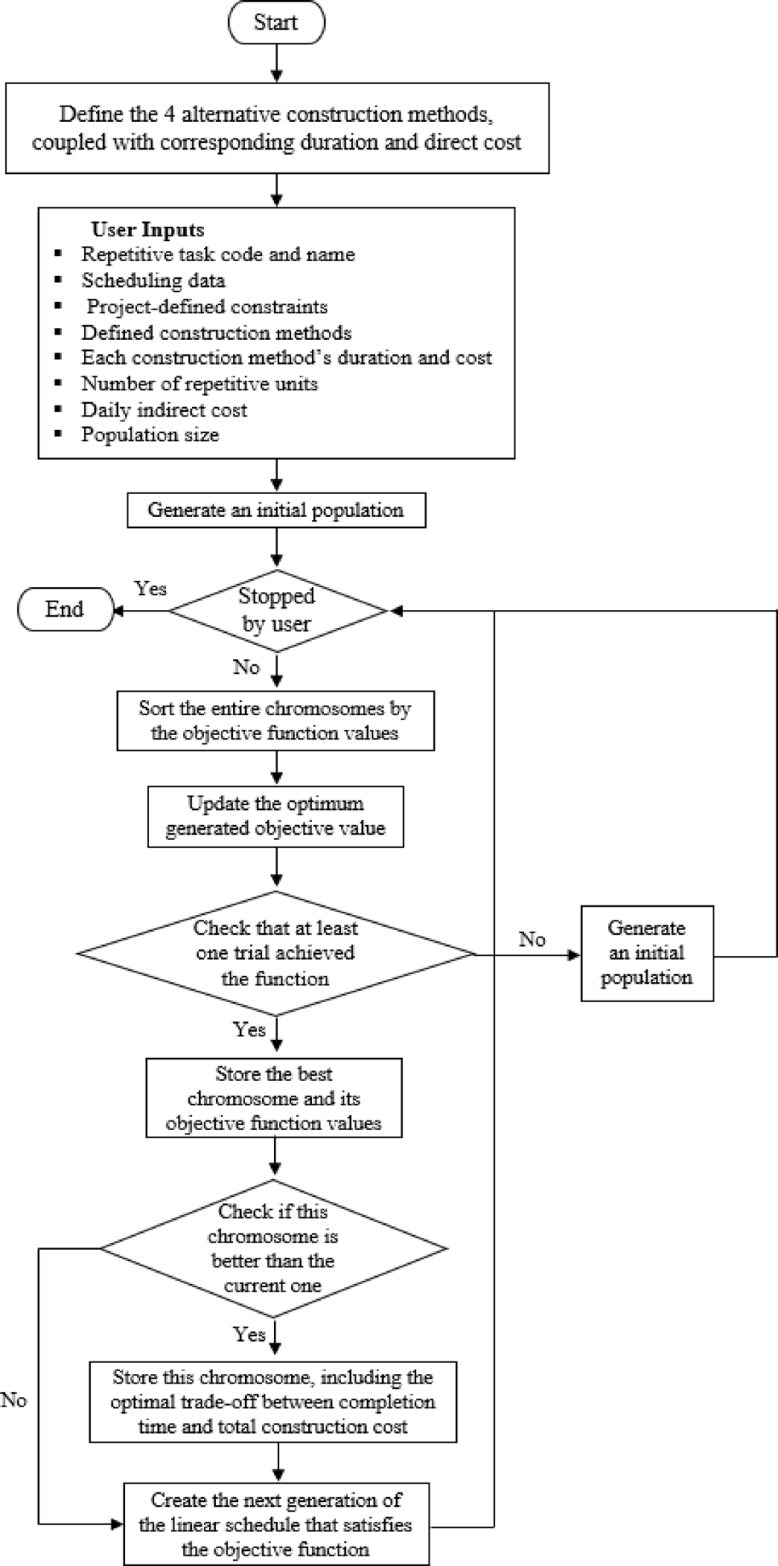
The proposed GA workflow.

To assess the effectiveness of the chromosome’s solution, the repetitive sub-tasks durations coupled with appointed direct costs are inferred, anticipating pertinent indices within the chromosome. The chromosome could depict arrays of linear project completion times. Each gene in the chromosome renders the start and end times for a particular repetitive sub-task, and the construction method is crucial to composing the repetitive sub-tasks. The chromosome comprises strings whose length displays the number of repetitive subtasks in the project. The intention of the optimization algorithm is thus to identify the chromosome that optimally trades off between time and cost, i.e., the chromosome that yields the shortest feasible completion time for the linear project while minimizing the total cost required to complete the repetitive sub-task. This process incorporates appraising the fitness of each chromosome in the population and using selection, mutation, and crossover operations to evolve the population over successive generations toward the optimal solution. Figure 5 exhibits the entire structure of the proposed GA approach.

#### Objective function

The pivotal objective function is minimizing the total linear repetitive project completion time F1, synchronously minimizing the total linear repetitive project costs F2, including direct and indirect costs, while employing predefined construction methods. The objective functions F1 and F2 are calculated based on the entire chromosomes per generation. Subsequently, the chromosomes are sorted, banking on the solution that attains the trade-off between minimizing F1 and concurrently minimizing F2. The objective functions are constituted and operated by changing the variable from 1 to 4, embodying the defined construction method for each repetitive sub-task. Followingly, the optimization model is subjected to these outlined constraints: (1) Decision variables are greater than or equal to 1 and less than or equal to 4, (2) Values of decision variables should be an integer, (3) The optimized total linear repetitive project cost should be less than the planned cost, and (4) The optimized linear repetitive project completion time should be less than the planned time.

#### Initial population engendering susceptible to the objective functions

Once the chromosome structure and fitness function are defined, the genetic algorithm begins the evolutionary optimization process. An initial population of N viable chromosomes is generated and ranked based on fitness. The population size, which the user can set, significantly influences both the solution quality and computation time—larger populations offer broader search capabilities but increase processing time.

Each chromosome’s fitness is evaluated, and the top-performing individuals are retained for reproduction. These elite chromosomes are carried forward to the next generation and participate in creating offspring through crossover and mutation. This evolutionary process enables the algorithm to refine solutions iteratively, optimizing the trade-off between project duration and cost.

**Fig. 6:**
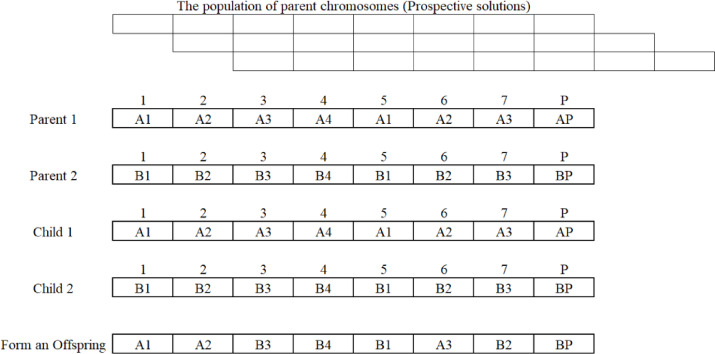
Crossover operator for creating offspring genes.

A single-point crossover operator is used during reproduction. Two parent chromosomes are selected based on fitness, and a crossover point is randomly chosen. The offspring inherit genes from one parent up to this point and from the second parent thereafter. This method preserves valuable genetic sequences while introducing diversity. Figure 6 demonstrates the single-point crossover operator, where two parent chromosomes, each representing a sequence of construction methods, exchange segments at a random crossover point. The resulting offspring combines parts of both parents, maintaining feasible scheduling sequences. This visual clarifies how the algorithm explores new solutions by varying method assignments across repetitive sub-tasks.

#### Genetic operators

Consequently, reproduction among population members emanates through crossover or mutation, which simulates natural evolution as genetic operators. Crossover is the more predominantly adopted process and involves picking two parent chromosomes, swapping relevant information, and engendering offspring. The selection of parent chromosomes is random but weighted by their relative merit, ensuring that the best chromosomes are more likely to be chosen while maintaining diversity. The process of exchanging genetic information between parent chromosomes is random in nature. Unlike crossover, which portrays natural reproduction, mutation exhibits a sparse process that can produce sudden, exceptional offspring. It involves randomly selecting a chromosome from the population and performing arbitrary evolutions on its information. As such, the mutation process can break any stagnation in the evolutionary process and avoid local minima.

In Fig. 6, assuming that two parents have been elected for engendering two offspring, the crossover operator partitions the parents’ sub-strings into two distinctive groups, relying on construction methods and substitutes the genes within each group’s sub-strings. As a result, the children derive the sequence of construction methods from both parents. Subsequently, these new children are translated into a feasible completion time and minimized total cost by decoding them.

After generating offspring using any of the available techniques, it undergoes a fitness evaluation. It can only be confined if it surpasses the other population members in terms of fitness. Typically, this cycle persists for numerous generations of offspring until a chromosome that represents the optimal solution is found. The current deployment allows the user to determine the number of generations of offspring as a discontinuity benchmark for the process. In addition, it is feasible for the user to halt the genetic operators when a satisfactory solution that meets the required criteria for the total project cost and time of completion is attained. In such a scenario, the output will present the total linear repetitive completion time, total project cost, and corresponding construction methods. Conversely, if the desired solution is not achieved, the algorithm will persist in probing for improved output.

The GA approach for LRPTCT is implemented using an optimization engine platform named Evolver TM Version 7. Evolver Palisade uses a GA approach to optimization, which involves using a population of potential solutions to a problem that undergoes a series of genetic operations, such as selection, crossover, and mutation, to generate new and potentially better solutions. The process is repeated over several generations until an optimal or near-optimal solution is obtained.

### Particle swarm optimization (PSO) workflow for LRPTCT

PSO represents a heuristic optimization technique inspired by the social behavior of organisms such as bird flocking and fish schooling. This method aims to find optimal solutions by mimicking the collaborative behavior of a swarm of particles searching for the best outcomes. In the context of LRPTCT, PSO provides a structured framework for minimizing project costs and durations by iteratively improving candidate solutions through collaboration and individual learning.

#### Encoding the particle and objective functions

**Fig. 7:**
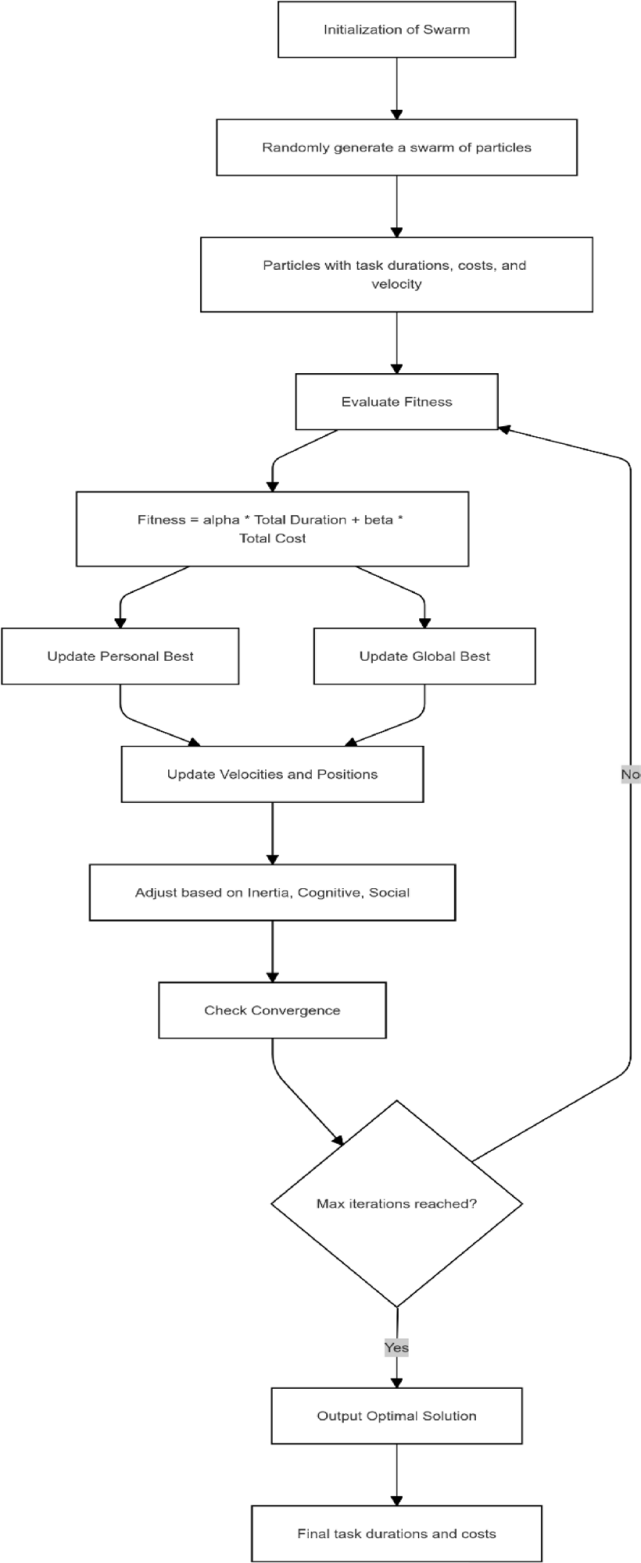
PSO workflow.

In the PSO framework (see Fig. 7), each particle represents a potential solution to the optimization problem. A particle is encoded as an array of decision variables, where each variable corresponds to the construction method selected for a repetitive task. The decision variables take integer values between 1 and 4, representing four predefined construction methods for each task. A particle’s position in the solution space denotes a unique configuration of selected methods evaluated based on fitness metrics. These metrics account for the total direct costs, indirect costs, and project duration associated with the methods chosen.

The PSO model for LRPTCT employs two objective functions. The first objective is to minimize the total project duration (F1), which is governed by the sequencing and dependencies of tasks, with critical tasks determining the overall duration. The second objective is to minimize the total project costs (F2), comprising Total Direct Costs (TDC) and Total Indirect Costs (TIC). TDC is derived from the costs of the construction methods chosen for each task, while TIC is calculated by multiplying the project duration by the daily overhead rate. These objective functions ensure the PSO model seeks an optimal trade-off between minimizing time and costs.

#### PSO optimization workflow

The PSO process for LRPTCT is structured into iterative steps, illustrating the PSO workflow. The workflow of the PSO framework begins with the initialization of a swarm of particles. Each particle is assigned a random position within the solution space, representing an initial configuration of construction methods. The initial velocities of the particles are set to zero. The fitness of each particle is evaluated using the objective functions, and the best-known positions for each particle (p_best) and the global best position (g_best) among all particles are recorded.

The iterative optimization process involves updating the velocity and position of each particle. The velocity update (Eq. 7) incorporates three components: the particle’s inertia, its cognitive tendency to return to its best-known position, and its social tendency to move toward the global best position. These updates are influenced by inertia weight (ω), cognitive coefficient (c1​), social coefficient (c2​), and random factors (r1​ and r2​) that add stochasticity to the search. After updating velocities, the positions of particles are adjusted to reflect the new configurations of construction methods. The positions are constrained within the valid range of decision variables, ensuring that all selected methods are feasible.

7 $${\text{V}}_{{{\text{i,t + 1}}}} = \omega {\text{v}}_{{{\text{i,t}}}} {\text{c}}_{{\text{1}}} {\text{r}}_{{\text{1}}} ({\text{p}}_{{{\text{best}}}} - {\text{p}}_{{{\text{i,t}}}} ) + {\text{c}}_{{\text{2}}} {\text{r}}_{{\text{2}}} ({\text{g}}_{{{\text{best}}}} - {\text{p}}_{{{\text{i,t}}}} )$$

Fitness values are recalculated for all particles after each update. If a particle’s new position improves upon its previous best-known position, its p_best is updated. Similarly, if a particle achieves a global improvement, g_best is updated. This iterative process continues until a stopping criterion is met, such as reaching a maximum number of iterations or achieving convergence to an optimal solution.

The PSO framework incorporates constraints to ensure practical and feasible solutions. Decision variables must remain integers between 1 and 4, representing valid construction methods. The optimized total project cost must be less than the planned budget, and the total project duration must not exceed contractual deadlines. Additionally, task dependencies and logical sequences are maintained throughout the optimization process to ensure constructability.

Upon completion, the PSO model outputs the optimal construction methods for all repetitive tasks, the minimized total project costs, the shortest feasible project duration, and detailed task schedules, including start and finish times. This comprehensive output supports decision-making in construction project management by providing actionable cost and time optimization insights.

The PSO framework demonstrates several advantages in addressing LRPTCT problems. It is highly scalable, accommodating projects with numerous tasks and complex dependencies. The algorithm’s iterative nature enables efficient convergence to optimal solutions, balancing exploration and exploitation to avoid local minima. Furthermore, PSO’s flexibility allows it to handle multi-objective optimization scenarios, making it suitable for minimizing costs and durations simultaneously.

## Developed approach implementation

**Fig. 8:**
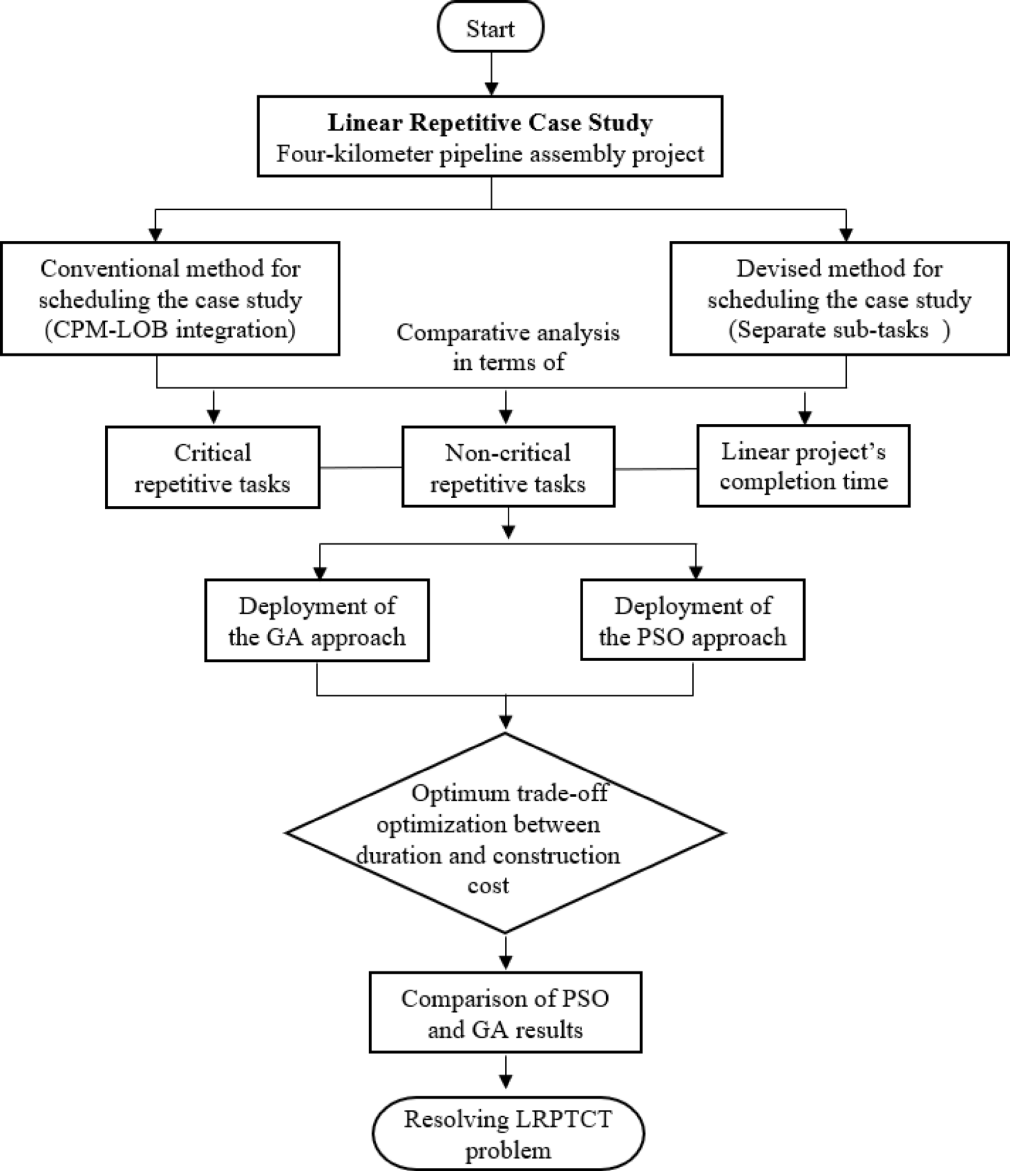
A Flowchart depicting the case study workflow.

To evaluate the effectiveness, reliability, and validity of the developed approaches, a case study was conducted using a four-kilometer pipeline assembly, as shown in Table 2. The developed methods are particularly suitable for horizontal infrastructure projects. The project consists of four parallel repetitive pipelines, each spanning 4 km, resulting in a total pipeline installation length of 16 km. The project’s precedence relations follow a Finish to Start (FS) sequence without lag time. The implementation process of the proposed approaches in the actual case study is illustrated in Fig. 8. Furthermore, the case study will include a comparative analysis between the proposed methods and the traditional CPM integrated with LOB to determine their overall project scheduling and cost management performance. This comparative analysis will provide a comprehensive understanding of the strengths of each approach.

## Results and discussion

### The conventional method for tackling the linear project’s scheduling

Scheduling the pipeline (4 km pipeline) for one unit unveiled eight critical tasks (A, B, C, D, F, G, K, and L) and four non-critical tasks (E, H, I, and J). According to the CPM results, the project’s duration for one unit is estimated to be 595 days, as rendered in Table 2. In Table 2, ES depicts the early start, EF is the early finish, LS is the late start, LF represents the late finish, and TF is the total float.

**Table 2:** Conventional CPM calculations for a single unit.

Tasks		Duration (Days)	Predecessors	Successors	ES	EF	LS	LF	TF
Code	No.								
A	1	14	-	2	0	14	0	14	0
B	2	7	1	3,8	14	21	14	21	0
C	3	112	2	4,5	21	133	21	133	0
D	4	140	3	6	133	273	133	273	0
E	5	112	3	6	133	245	161	273	28
F	6	252	4,5	7	273	525	273	525	0
G	7	28	6	11	525	553	525	553	0
H	8	56	2	9	21	77	343	399	322
I	9	119	8	10	77	196	399	518	322
J	10	35	9	11	196	231	518	553	322
K	11	14	7,10	12	553	567	553	567	0
L	12	28	11	FN	567	595	567	595	0
	FN	0	12		595	595	595	595	0

### The devised method for addressing the linear project’s scheduling

To more accurately reflect practicability in linear repetitive construction projects, the tasks previously mentioned were divided into separate sub-tasks of equal length. Each sub-task was treated as a flexible task with a length of 1 km and a duration equal to the total duration of the original task divided by 4. This paradigm was designed to establish a relationship between the previously separate tasks, unearthing more feasible completion times. Table 3 displays the case study results after splitting tasks C, D, E, F, and G into four equal 1-kilometer sub-tasks. The reduction of single-unit duration is evident. The total duration decreased by 210 days (depicting a 35% reduction) from 595 days, computed using the traditional CPM method, to 385 days after the tasks were split, as explicitly portrayed in Tables 2 and 3. Apropos of Table 3, the division of tasks C, D, and G revealed the presence of partially non-critical sub-tasks. Specifically, only C1 is rendered a critical task within task C, whereas non-critical tasks represent C2, C3, and C4. Likewise, D1 is the sole crucial sub-task within task D, while D2, D3, and D4 are categorized as non-critical. Finally, in task G, only G4 is considered critical, while G1, G2, and G3 are classified as non-critical sub-tasks.

To compute the required number of crews for each sub-task, the computation of the desired delivery rate (Rd) for the repetitive units is initially undertaken using Eq. 8. In Eq. 8, variables are assigned as follows: n signifies the number of repetitive units, TL denotes the project’s deadline duration as stipulated in the contract agreement, T1 depicts the CPM duration of the first unit, and Tfi embodies the total float of each sub-task. Subsequently, the estimation of the number of crews required to perform each sub-task efficiently is attained by employing Eq. 9. In Eq. 8, Ci denotes the number of crews assigned to each sub-task (i), and Ri embodies the desired delivery rate for each sub-task (i). Therefore, the number of crews entailed by each sub-task may vary depending on each sub-task’s Rd, Tfi, and designated duration.

8 $${\text{Ri}} = \left( {{\text{n }}{-}{\text{ 1}}} \right)/\left( {{\text{TL }} - {\text{ T1}}} \right) + {\text{Tfi}}$$

9 $${\text{Ci}} = {\text{Di}} \times {\text{Ri}}$$

**Table 3:** Scheduling calculations for a single unit, considering splitting tasks into sub-tasks.

Tasks		Duration (Days)	Predecessors	Successors	ES	EF	LS	LF	TF
Code	No.								
A	1	14	-	2	0	14	0	14	0
B	2	7	1	3,23	14	21	14	21	0
C1	3	28	2	4,7,11	21	49	21	49	0
C2	4	28	3	5,8,12	49	77	84	112	35
C3	5	28	4	6,9,13	77	105	147	175	70
C4	6	28	5	10,14	105	133	210	238	105
D1	7	35	3	8,15	49	84	49	84	0
D2	8	35	4,7	9,16	84	119	112	147	28
D3	9	35	5,8	10,17	119	154	175	210	56
D4	10	35	6,9	18	154	189	238	273	84
E1	11	28	3	12,15	49	77	56	84	7
E2	12	28	4,11	13,16	77	105	119	147	42
E3	13	28	5,12	14,17	105	133	182	210	77
E4	14	28	6,13	18	133	161	245	273	112
F1	15	63	7,11	16,19	84	147	84	147	0
F2	16	63	8,12,15	17,20	147	210	147	210	0
F3	17	63	9,13,16	18,21	210	273	210	273	0
F4	18	63	10,14,17	22	273	336	273	336	0
G1	19	7	15	20	147	154	315	322	168
G2	20	7	16,19	21	210	217	322	329	112
G3	21	7	17,20	22	273	280	329	336	56
G4	22	7	18,21	26	336	343	336	343	0
H	23	56	2	24	21	77	133	189	112
I	24	119	23	25	77	196	189	308	112
J	25	35	24	26	196	231	308	343	112
K	26	14	22,25	27	343	357	343	357	0
L	27	28	26	FN	357	385	357	385	0
	FN	0	27		385	385	385	385	0

**Fig. 9:**
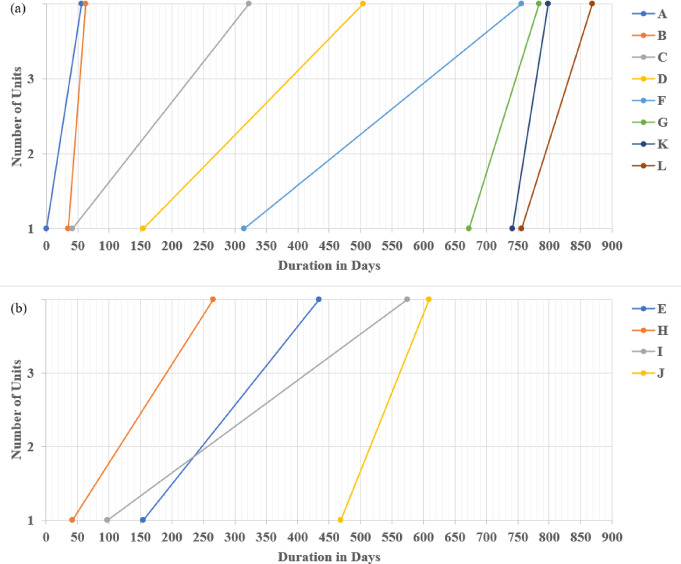
LOB depiction using traditional CPM calculations. (**a**) Critical path showing critical repetitive tasks; (**b**) LOB diagram of non-critical tasks.

Using traditional CPM calculation, rendering the four km pipeline assembly using the Line Of Balance (LOB) scheduling method for the repetitive units unveiled the linear repetitive project’s duration as 868 days. A, B, C, D, F, G, K, and L donate the critical linear repetitive tasks, whereas E, H, I, and J portray the non-critical repetitive tasks, as exhibited in Fig. 9.

**Fig. 10:**
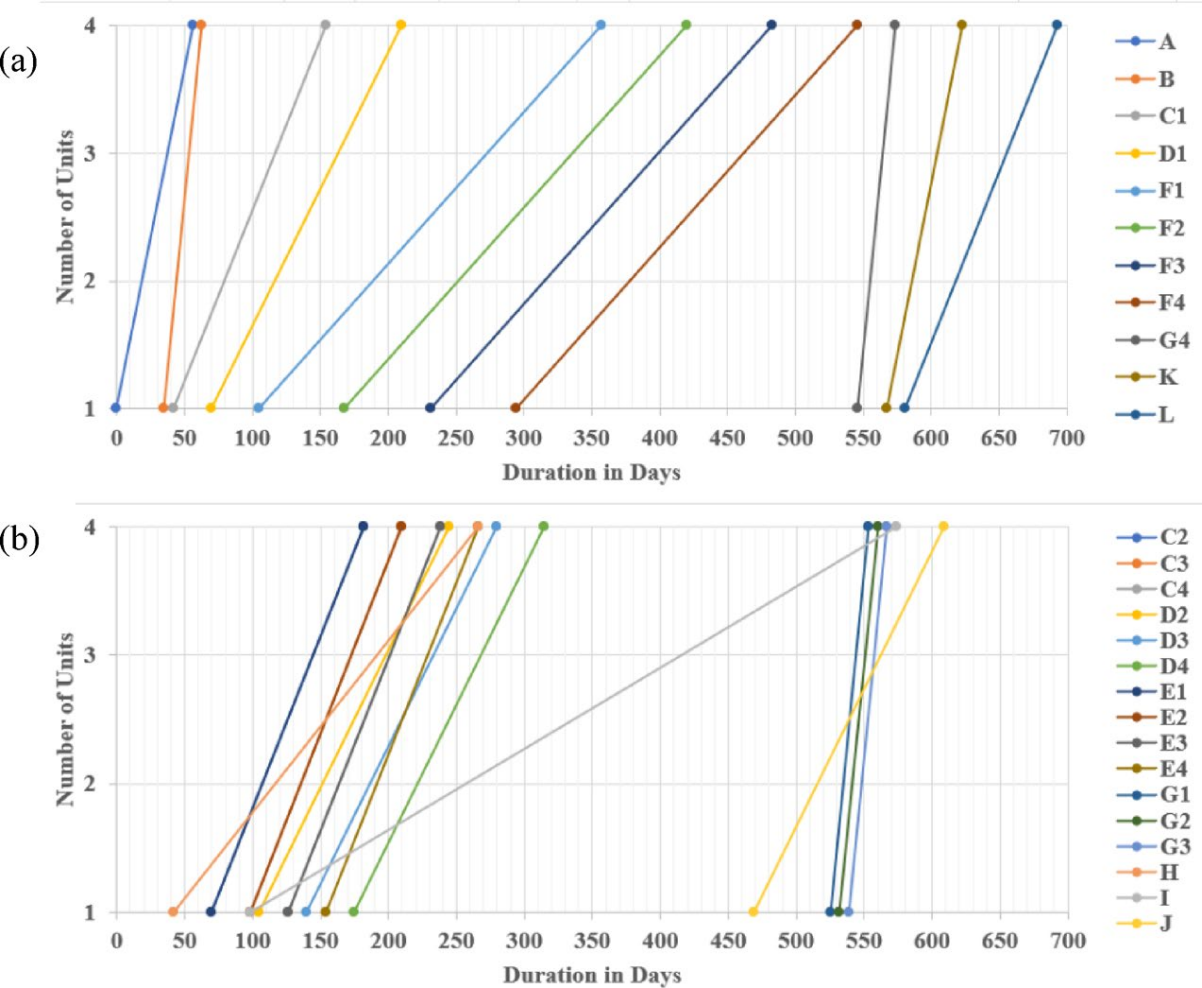
LOB depiction using the proposed approach for splitting into sub-tasks. (**a**) Critical path embodying critical repetitive sub-tasks; (**b**) LOB diagram of non-critical sub-tasks.

The data portrayed in Fig. 10 corroborates the outcome of implementing the LOB scheduling method for repetitive units in rendering the 4 km pipeline installation. This was achieved by dividing tasks C, D, E, F, and G into four equal sub-tasks of 1 km each. Consequently, the linear repetitive project was reduced, reducing the total duration from 868 days using the traditional LOB-integrated CPM method to 693 days (exhibiting nearly a 20% reduction). The critical repetitive tasks were identified as A, B, C1, D1, F1, F2, F3, F4, G4, K, and L. In contrast, the non-critical repetitive tasks were C2, C3, C4, D2, D3, D4, E1, E2, E3, E4, G1, G2, G3, H, I, and J. Accordingly, the approach of considering non-critical sub-tasks in linear repetitive projects is vital for achieving a balance between minimizing project costs and time. These non-critical sub-tasks, including C2, C3, C4, D2, D3, D4, G1, G2, and G3, can reduce the number of employed crews and relax the activity production rate, thereby empowering the prospect of optimizing the LRPTCT. It is worth mentioning that non-critical activities play an essential role in scheduling repetitive projects, contributing to the overall efficiency and successful project delivery. For instance, non-critical activities provide opportunities to employ accessible resources effectively. Hence, when critical activities do not consume all resources, crews can be assigned to non-critical activities, maximizing their productivity and avoiding idle time.

Further, Non-critical activities maintain a continuous workflow, particularly during delays or disruptions in critical activities. The project can progress steadily by assigning crews to non-critical tasks even if certain critical activities are behind schedule, promoting a smooth flow of work and preventing unnecessary downtime. Accordingly, this allows project managers to respond effectively to unexpected circumstances and maintain project momentum.

The LOB diagram depicted in Fig. 10 exhibits a distinctively consecutive progression of activities, whereby each step constitutes a direct connection with the subsequent one, devoid of interruptions or temporal intervals. This discernible attribute stems from the underlying premise of zero buffer time among the activities, guaranteeing an expeditious execution of the linear schedule. However, it is crucial to highlight that incorporating buffer time between activities can constitute a valuable scheme to accommodate unforeseen delays or variations in scheduling linear projects.

### Deployment of the GA approach

After the completion of the GA model, an initial evaluation was carried out to identify appropriate GA parameter values, such as population size and the number of generations. Apropos of the research conducted ^35^ and ^34^, a population size of 100 and 1000 generations was deemed a satisfactory trade-off between diversity and processing time. For lessening computational load, a set of algorithm attribute values was presented, encompassing (1) the iteration rate is 60%, (2) the crossover likelihood is 26%, (3) the mutation likelihood is 10%, and (4) the migration likelihood is 9%. The chromosome comprises strings whose length is 27, portraying the number of repetitive sub-tasks in the linear repetitive project. Table 4 exhibits the cost and duration information for the construction methods of repetitive tasks and sub-tasks. The intended direct cost of the 27 repetitive tasks was deployed as the initial cost, which led to a total cost of 45,617,920 EGP and a completion period of 693 days for all units. According to the project’s contractual agreements, the agreed-upon indirect cost is estimated as a daily fixed value of 3,000 EGP, which is typically discerned by breaking down the various components of indirect costs, such as site overhead and general overhead. However, the fixed value of indirect costs can vary depending on multiple factors, including the nature of the project, industry practices, and the specific agreement between the owner and the contractor. Consequently, the project’s total cost is approximated at 56,621,920 EGP.

**Fig. 11:**
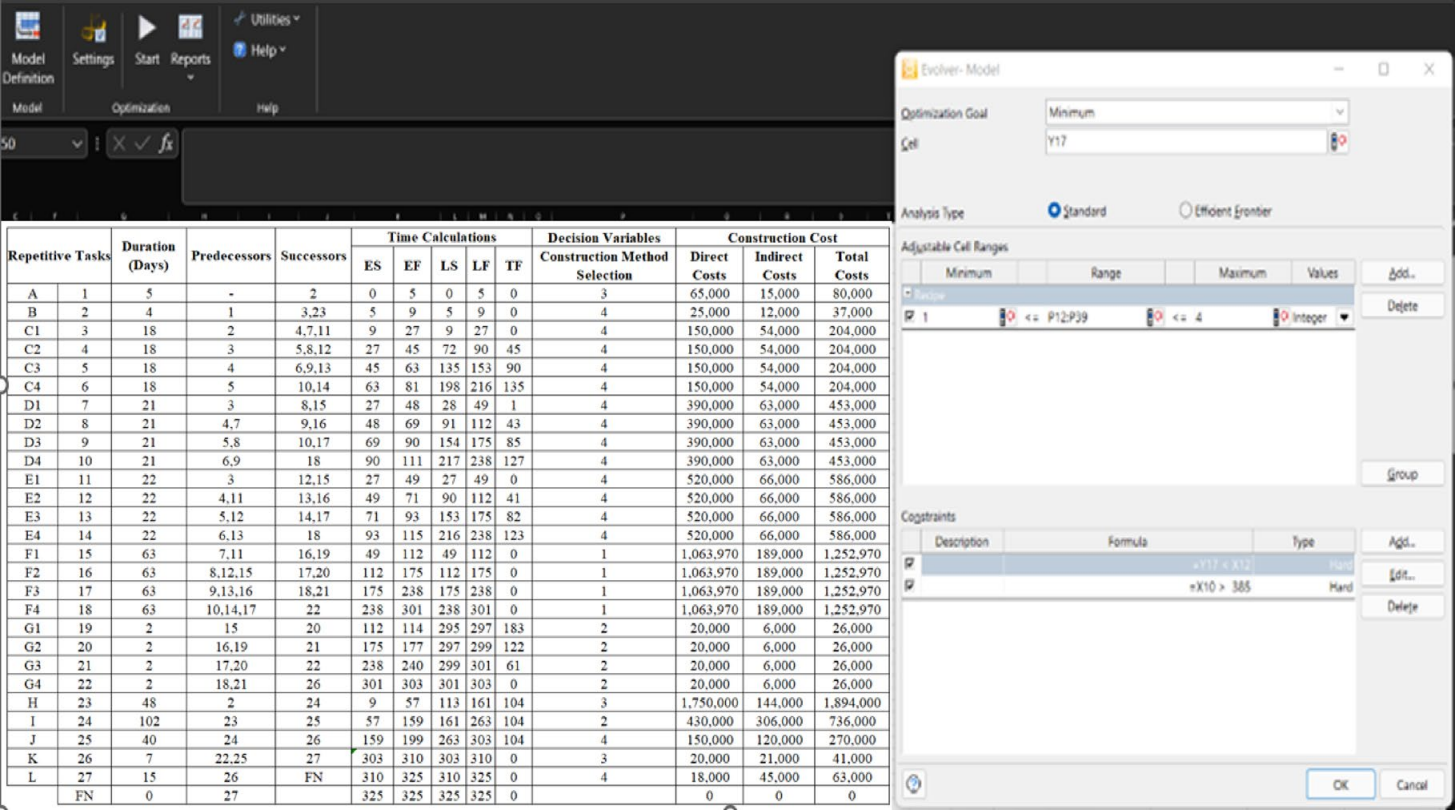
The formulation of the GA model for LRPTCT using the optimization engine.

To this end, the inputs to the GA model are (1) Each repetitive task code and name, (2) scheduling data, including predecessors and successors of each repetitive task, (3) different alternatives of construction methods, incorporating each method’s direct cost and designated duration, (4) the project defined constraints as previously outlines, and (5) the number of repetitive units and daily indirect cost for each repetitive activity, as displayed in Fig. 11.

**Table 4:** Construction methods alternatives depicting the GA model’s decision variables.

Construction Method #	Method #1		Method #2		Method #3		Method #4	
Repetitive tasks	Duration (Days)	Cost (EGP)	Duration (Days)	Cost (EGP)	Duration (Days)	Cost (EGP)	Duration (Days)	Cost (EGP)
A	14	40,400	10	60,000	5	65,000	12	53,000
B	7	18,550	1	50,000	3	35,000	4	25,000
Each sub-task C	28	137,200	14	200,000	20	170,000	18	150,000
Each sub-task D	35	353,500	25	380,000	18	450,000	21	390,000
Each sub-task E	28	594,000	40	510,000	20	680,000	22	520,000
Each sub-task F	63	1,063,970	55	1,120,000	30	1,350,000	45	1,250,000
Each sub-task G	7	8,400	2	20,000	5	12,000	10	10,000
H	56	2,003,700	70	2,750,000	48	1,750,000	35	2,150,000
I	119	499,850	102	430,000	85	580,000	90	620,000
J	35	174,500	18	220,000	25	200,000	40	150,000
K	14	26,600	10	40,000	7	20,000	4	45,000
L	28	12,600	18	16,000	26	10,000	15	18,000

**Fig. 12:**
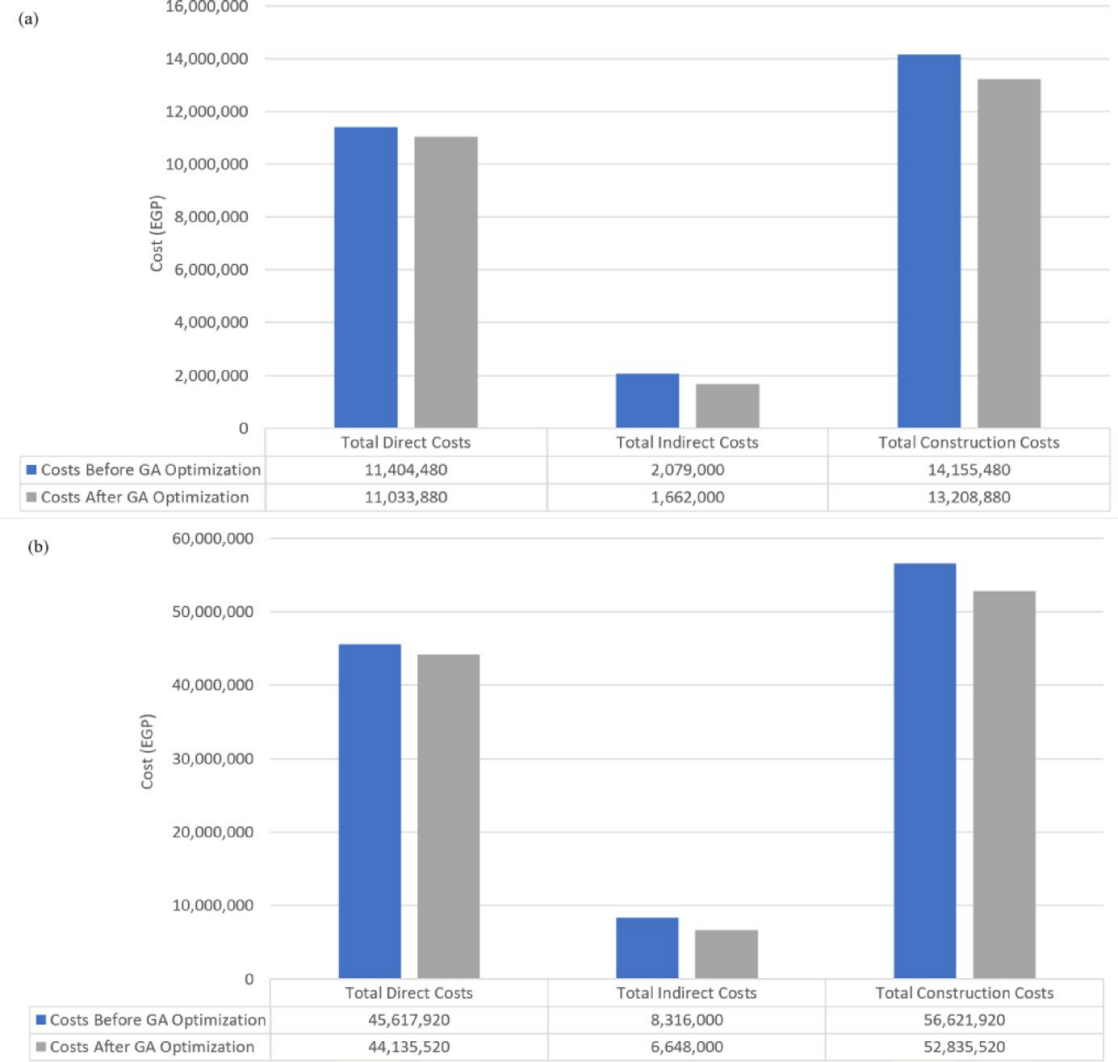
Comparison of the developed GA with the base case apropos of costs. (**a**) Linear project costs comparison for one linear unit; (**b**) Linear project costs comparison for the four repetitive units.

According to the data presented, the proposed model underwent 100,000 trials, of which 77,820 were deemed valid. The computations and running time of the model were performed using an Intel Core i7 computer and lasted approximately 10 min. After running the optimization engine, the GA-proposed approach results revealed a reduction in the linear project’s TDC, TIC, and Total Construction Cost (TCC) apropos of one linear unit from 11,404,480 EGP to 11,033,880 EGP, from 2,079,000 EGP to 1,662,000 EGP, and from 14,155,480 EGP to 13,208,880 EGP, respectively (as shown in Fig. 12). Corrpsonigly, the GA-proposed approach unearthed a reduction in the linear repetitive project TDC, TIC, and TCC apropos of the four linear repetitive units from 45,617,920 EGP to 44,135,520 EGP, from 8,316,000 EGP to 6,648,000 EGP, and from 56,621,920 EGP to 52,835,520 EGP, progressively, as rendered in Fig. 12. The findings indicate that the GA-proposed approach successfully decreased the linear repetitive project’s TDC, TIC, and TCC by approximately 3.25%, 20%, and 7%, respectively.

**Fig. 13:**
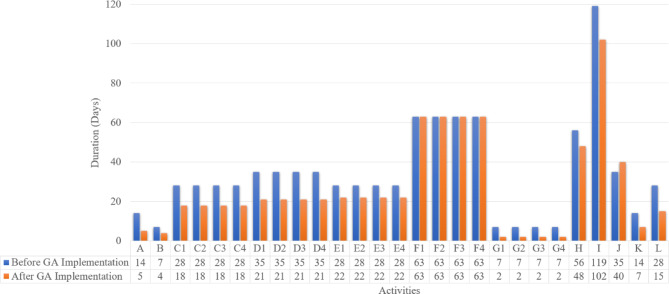
Comparison of the developed GA with the base case apropos of the duration per each repetitive task.

Regarding the time optimization, the GA-proposed approach results revealed a reduction in the linear repetitive project’s completion time pertinent to one unit and four units from 385 to 325 and 693 to 554, enlightening approximately 16% and 20% reduction, respectively. Figure 13 portrays a comparative analysis between the developed GA and the base case with respect to the duration of each repetitive task. The stupendous reduction in TDC, TIC, TCC, and completion time of the linear repetitive project corroborates and supports the developed approach, achieving a tremendous reduction in performance. These reductions are attributable to the proposed approach of exhibiting the repetitive tasks as a series of distinct sub-tasks interconnected to render the overall duration of the task. This approach reckoned with depicting any logical connection between the decomposed tasks, showing more methodical schedules, and portraying non-critical sub-tasks. These non-critical sub-tasks in linear repetitive projects are vital for balancing project costs and time, thus empowering the prospect of optimizing the LRPTCT. This hypothesis can be explicitly depicted in Fig. 13, rendering an immense reduction in non-critical sub-tasks duration due to deploying their total floats constituted from decomposing the repetitive tasks.

**Fig. 14:**
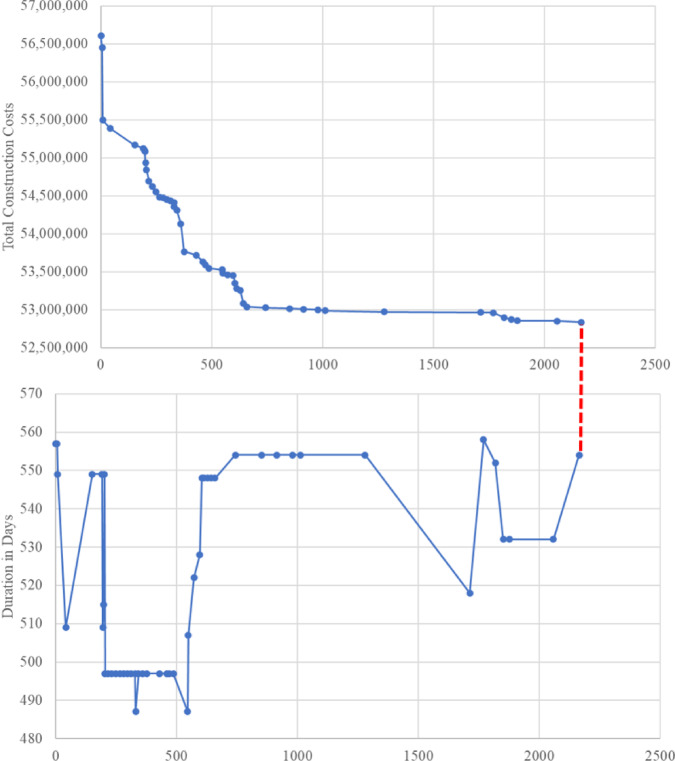
The optimum trade-off optimization between the total project’s duration and construction cost.

**Fig. 15:**
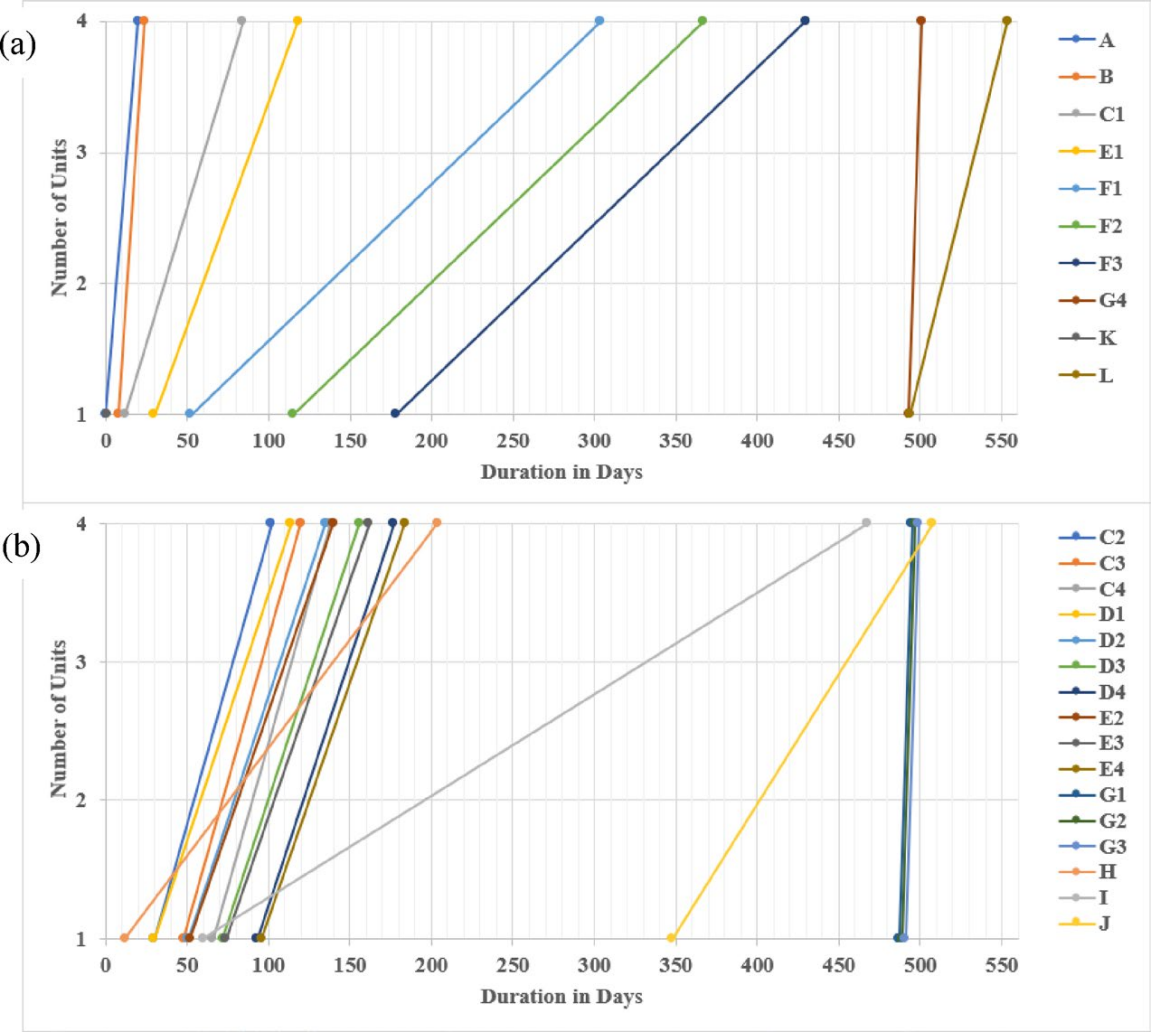
LOB depiction after deploying the GA optimization approach. (**a**) Critical path embodying critical repetitive sub-tasks; (**b**) LOB diagram of non-critical sub-tasks.

Figure 14 renders the optimum trade-off optimization between the total project’s duration and construction cost attained from the GA trials, unveiling the number of trials carried out by the GA platform to engender the optimum outcome. It can be inferred from Fig. 14 that the best trial number to attain the optimum results was 2,165 trials. Ultimately, Fig. 15 exhibits the LOB depiction after deploying the proposed GA paradigm, unearthing the reduction in the linear repetitive project’s completion time pertinent to the four repetitive units from 693 to 554 (nearly 20% reduction in the completion time).

### Deployment of the PSO approach

The deployment of the PSO algorithm for optimizing linear repetitive project scheduling involved a rigorous evaluation of model parameters to ensure a balance between computational efficiency and solution quality. Based on established heuristic optimization methodologies, the PSO algorithm was configured with the following parameters: (1) Population Size: 30 particles representing potential solutions, each encoding the method selection for all repetitive tasks; (2) Number of Iterations: 100, ensuring sufficient exploration of the solution space; (3) Inertia Weight (ω): Set to 0.5, to balance exploration (searching for new solutions) and exploitation (refining existing solutions); (4) Cognitive Coefficient (c1​): 2, to emphasize a particle’s self-learning from its own best-known solution, (5) Social Coefficient (c2c​): 2, to promote collaboration among particles by gravitating toward the global best solution.

Each particle’s position represented a unique configuration of selected methods for 27 repetitive tasks. These tasks were derived from a linear repetitive construction project, and their respective costs and durations were provided across four construction method alternatives. During the initial configuration, the project incurred a TDC of 45,617,920 EGP and required 693 days for completion. Indirect costs were fixed at 3,000 EGP daily, leading to a TCC of 56,621,920 EGP. These values formed the baseline for evaluating the PSO model’s performance.

The input data required for the PSO model included: (1) Repetitive Task Details: Each task’s identifier and its precedence relationships with other tasks, ensuring logical task sequencing; (2) Construction Method Alternatives: Four potential methods for each task, each defined by its associated direct cost and duration; (3) Project Constraints: Included adherence to contractual deadlines, logical dependencies, and resource availability; (4) Daily Indirect Costs: Fixed at 3,000 EGP per day, contributing to overall project overheads, and (5) Number of Repetitive Units: The analysis considered four units of the repetitive project.

**Fig. 16:**
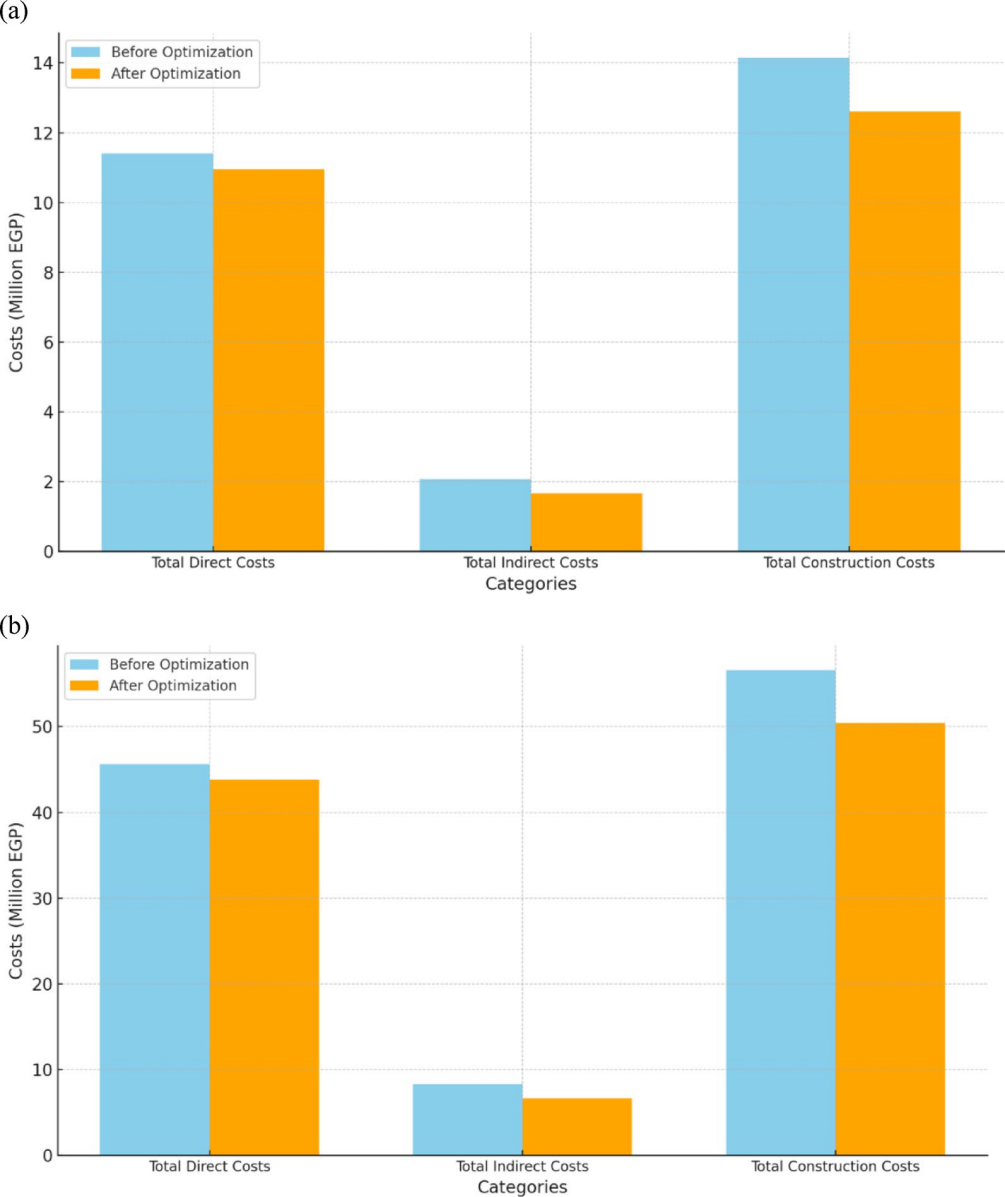
Cost comparison before and after optimization using PSO. (**a**) One unit; (**b**) Four units.

The cost optimization results of the PSO model demonstrated significant reductions across all major cost categories, including TDC, TIC, and TCC. For a single linear repetitive project unit, the TDC before optimization was 11,404,480 EGP, which reflected the baseline selection of construction methods without consideration for optimization. After applying the PSO model, the TDC was reduced to 10,947,910 EGP, achieving a 4.0% reduction. This improvement was primarily attributed to selecting cost-efficient construction methods for tasks and sub-tasks while maintaining adherence to project constraints and logical dependencies, as rendered in Fig. 16.

For the entire repetitive project consisting of four units, the TDC initially stood at 45,617,920 EGP. After optimization, it was reduced to 43,791,640 EGP, representing a 4.0% reduction. These consistent reductions across single and multi-unit analyses underscore the scalability of the PSO model in managing repetitive project costs effectively. By prioritizing lower-cost methods, the algorithm ensured that direct costs were minimized without negatively impacting other project parameters, as shown in Fig. 16.

The TIC, which depends on the project duration and daily overhead rates, exhibited a much larger reduction than the TDC. Before optimization, the TIC for one unit was 2,079,000 EGP, derived from a baseline project duration of 385 days and a daily indirect cost rate of 3,000 EGP. After optimization, the TIC was reduced to 1,662,000 EGP, reflecting a substantial 20.0% reduction. This reduction is directly linked to the model’s ability to shorten project duration, as indirect costs are proportional to time. The TIC was reduced from 8,316,000 EGP to 6,648,000 EGP for four repetitive units, maintaining the same 20.0% reduction rate (see Fig. 16). The efficiency of the PSO algorithm in compressing task durations while maintaining logical sequencing contributed significantly to these reductions.

The combined impact of TDC and TIC optimizations resulted in notable reductions in the TCC. For one unit, the TCC decreased from 14,155,480 EGP before optimization to 12,609,910 EGP after optimization, representing a 10.9% reduction. Similarly, the TCC decreased from 56,621,920 EGP to 50,439,640 EGP for four units, reflecting a 10.9% reduction. The dual emphasis of the PSO model on minimizing direct costs and reducing project duration proved effective in achieving these comprehensive cost savings. Figure 16 provides a comparative illustration of these reductions, clearly depicting the efficiency gains realized through the PSO model.

The Particle Swarm Optimization model also substantially improved project completion time for single-unit and multi-unit analyses. The baseline completion time for a single linear repetitive project unit was 385 days. After optimization, this was reduced to 315 days, reflecting an 18.2% reduction. This improvement was achieved by prioritizing faster construction methods for critical tasks while strategically assigning float times to non-critical sub-tasks. Composing repetitive tasks into sub-tasks enabled the PSO model to allocate resources more effectively, ensuring that time reductions were achieved without compromising project quality or logical sequencing.

**Fig. 17:**
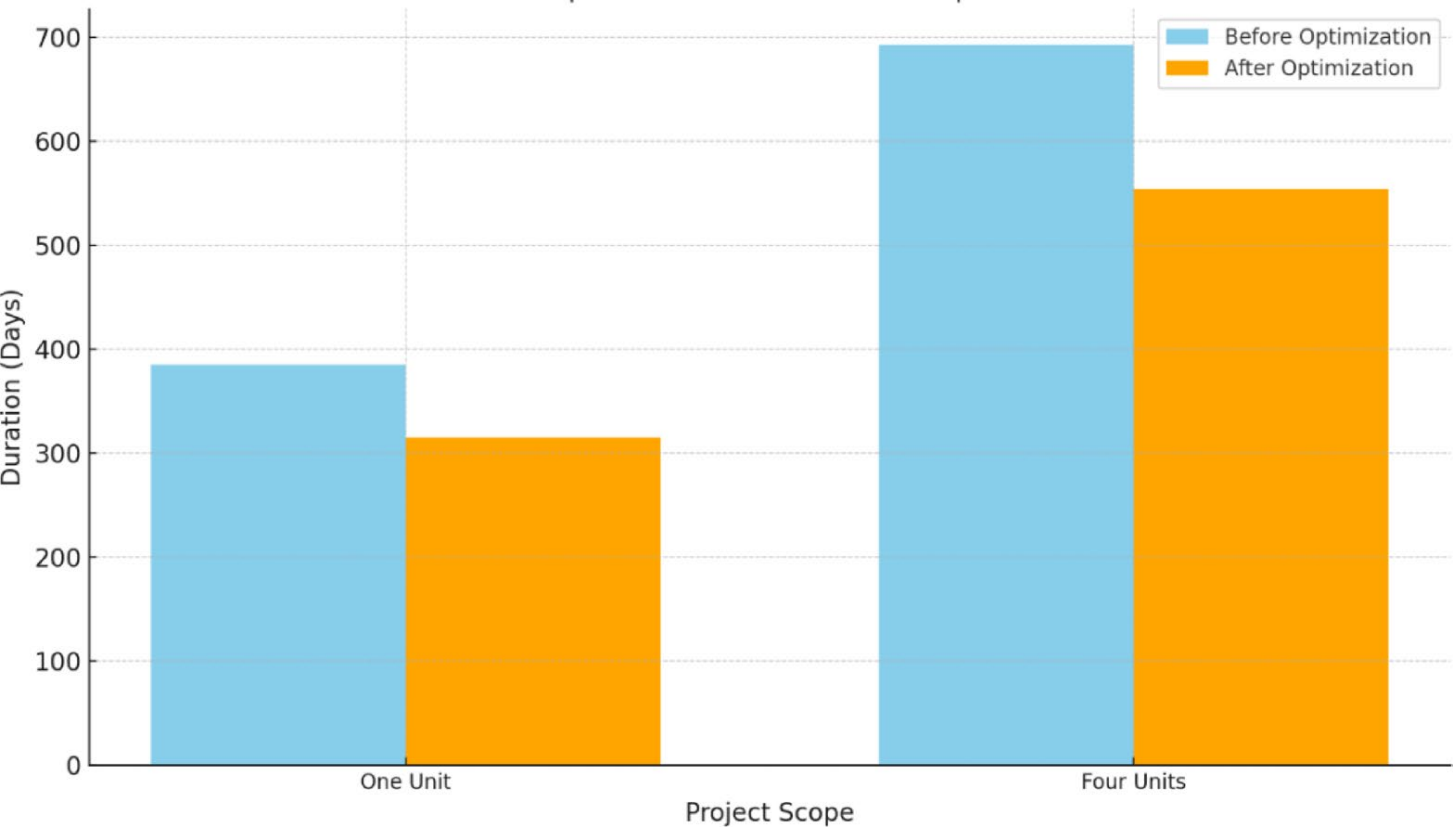
Time comparison before and after optimization using PSO.

For the multi-unit analysis involving four repetitive project units, the baseline completion time was 693 days. After applying the PSO model, the total completion time was reduced to 554 days, representing a 20.1% reduction. The larger time savings in the multi-unit scenario were due to the model’s ability to optimize non-critical tasks across all units simultaneously, effectively minimizing idle times and ensuring efficient resource utilization. By leveraging float times, the PSO model significantly reduced the duration of non-critical tasks, contributing to a streamlined project timeline while not directly impacting the critical path. These time savings are visually represented in Fig. 17, highlighting the reductions achieved for critical and non-critical tasks.

The optimization of project durations directly influenced the reduction in Total Indirect Costs, as shorter project durations translate to lower overhead costs. This interdependence of cost and time optimization underscores the strength of the PSO model in addressing the multi-dimensional nature of construction project management. The ability to balance the trade-offs between cost minimization and time compression makes the PSO model a valuable tool for optimizing linear repetitive projects.

### Comparison of PSO and GA results

**Fig. 18:**
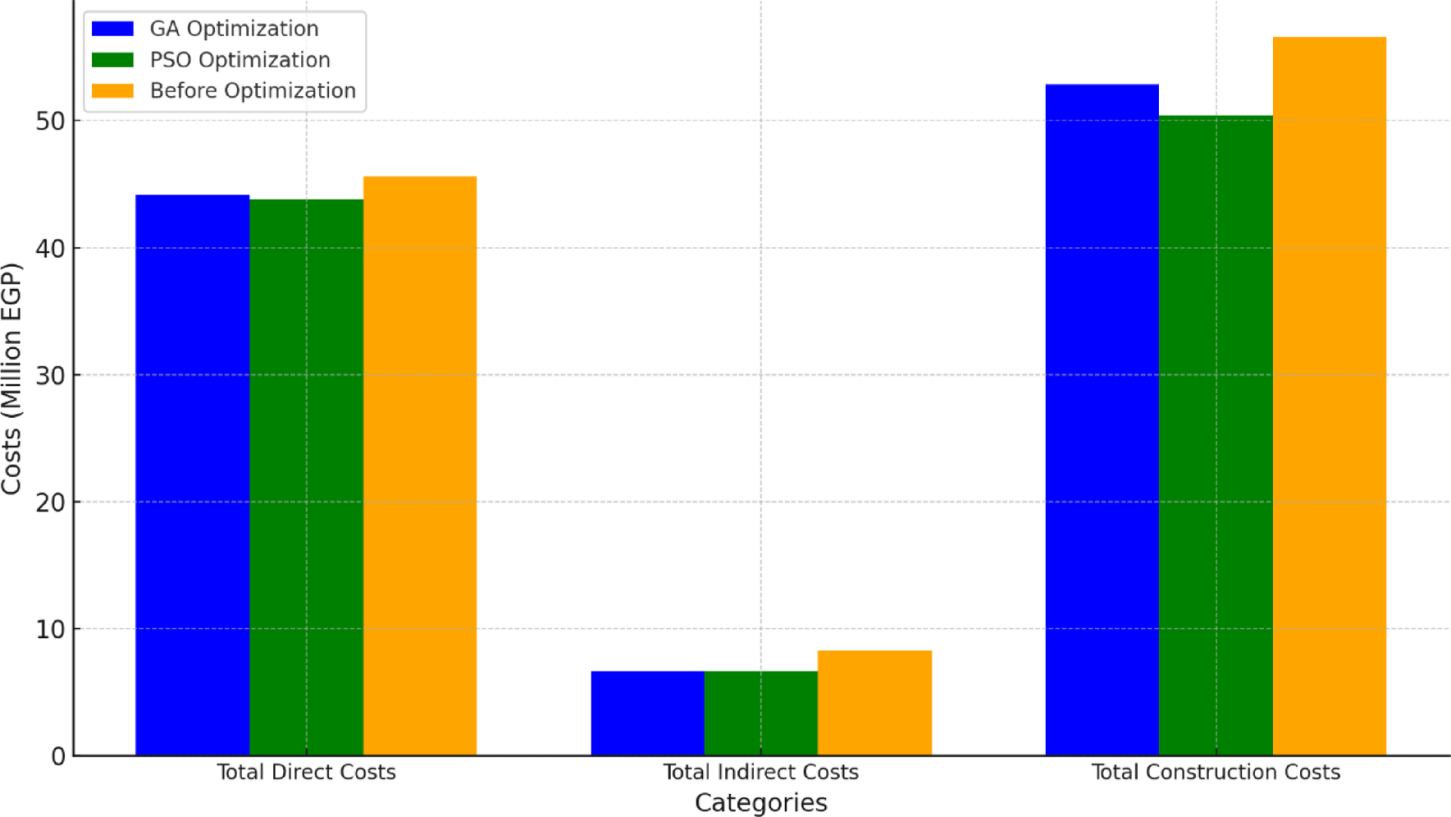
Cost comparison between GA and PSO for four units.

The PSO and GA models were applied to optimize costs and durations for a linear repetitive project. The comparison below provides a detailed analysis of their respective performances. Regarding cost optimization, for a single project unit, TDC was reduced by both methods, with PSO achieving a final TDC of 10,947,910 EGP compared to GA’s slightly higher TDC of 11,033,880 EGP. Both methods provided significant improvements over the baseline TDC of 11,404,480 EGP. Both PSO and GA demonstrated identical results for TIC, reducing the baseline TIC of 2,079,000 EGP to 1,662,000 EGP. Regarding the TCC, PSO outperformed GA by achieving a final TCC of 12,609,910 EGP, compared to GA’s TCC of 13,208,880 EGP, with both models achieving notable reductions from the baseline of 14,155,480 EGP (see Fig. 18).

The results of the multi-unit analysis, which comprised four repetitive units, followed a similar trend. PSO reduced the TDC to 43,791,640 EGP, while GA resulted in a TDC of 44,135,520 EGP, both improving on the baseline of 45,617,920 EGP. The TIC for the multi-unit project was reduced from the baseline of 8,316,000 EGP to 6,648,000 EGP by both PSO and GA. The TCC achieved by PSO was lower at 50,439,640 EGP, compared to GA’s TCC of 52,835,520 EGP, representing significant savings over the baseline of 56,621,920 EGP.

**Fig. 19:**
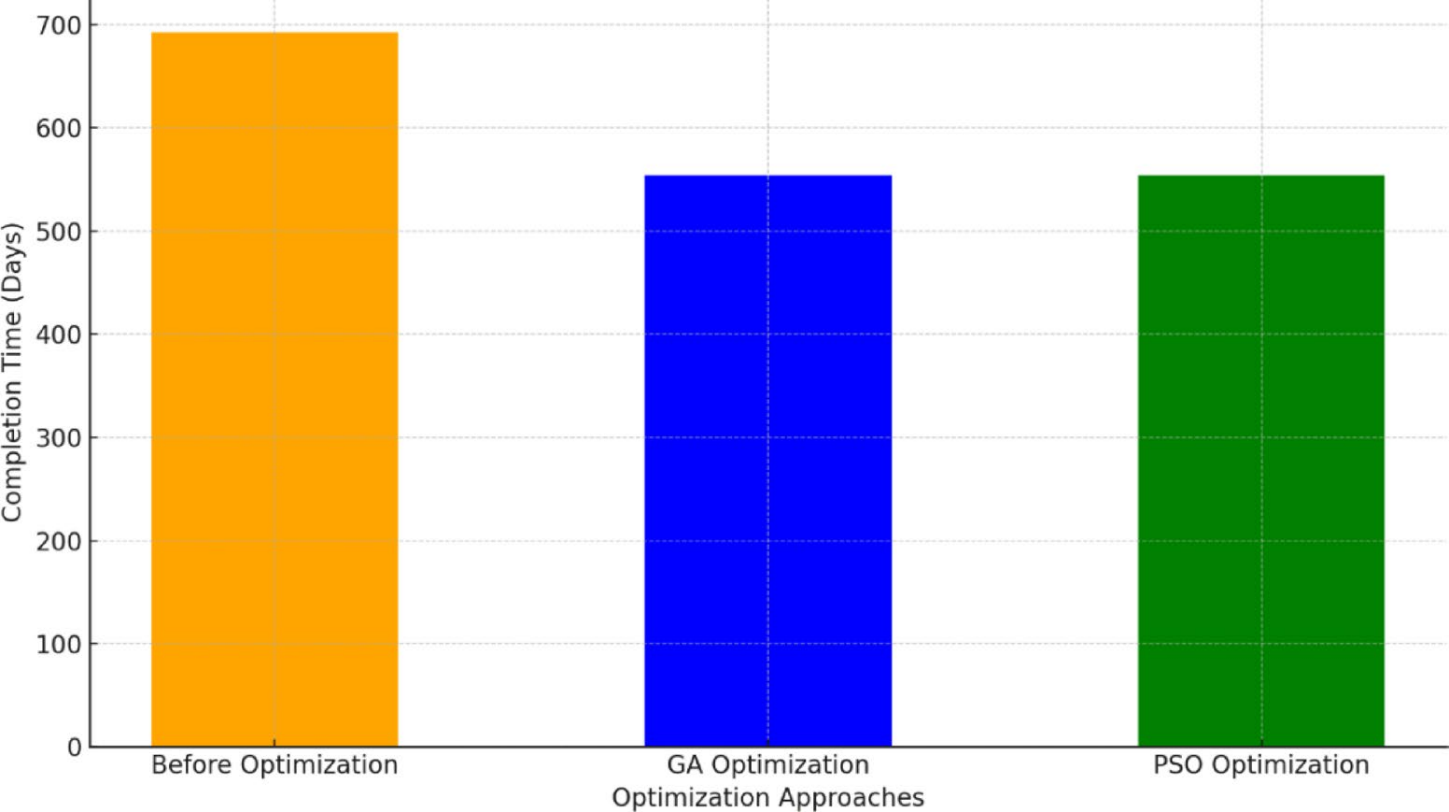
Time comparison between GA and PSO for four units.

Regarding time optimization, PSO reduced the single-unit project completion time from 385 days to 315 days, representing an 18.2% reduction. GA achieved a similar reduction, reducing the project duration to 325 days. For the multi-unit scenario, PSO reduced the project completion time from 693 days to 554 days, a 20.1% reduction. Similarly, GA reduced the completion time for four units to 554 days, matching PSO’s performance in this regard (Fig. 19). It is worth mentioning that the observed reductions in TDC and TIC translate directly into practical improvements on-site. Lower TDC values indicate that the optimization framework successfully selected more cost-efficient construction methods without compromising project logic, reducing material and labor expenditures. Similarly, reductions in TIC reflect shortened project durations, which minimize overhead costs such as site supervision, temporary facilities, and equipment rentals. These improvements suggest better crew coordination, less idle time, and more streamlined execution of repetitive tasks—key indicators of enhanced operational efficiency in linear construction projects.

The comparative analysis highlights that while PSO and GA excel in optimizing costs and time, PSO consistently outperformed GA in achieving lower Total Construction Costs for single-unit and multi-unit projects. Both methods delivered equivalent reductions in Total Indirect Costs and project completion times, indicating similar performance in addressing the time dimension. These results demonstrate the robustness of PSO and GA in solving complex optimization problems, with PSO showing a slight edge in cost efficiency.

It is worth mentioning that the parameter settings for GA and PSO were selected based on established literature and preliminary testing to balance solution quality and computational efficiency. A population size of 100 and 1,000 generations for GA, along with PSO’s inertia weight (0.5), cognitive (2.0), and social coefficients (2.0), were chosen to ensure convergence without excessive runtime. While these values were effective, a formal sensitivity analysis was not conducted, and results may vary with different parameter settings. This limitation highlights the need for future research to assess parameter sensitivity and optimize settings for different project conditions.

## Conclusions and prospective work

This study introduced a metaheuristic-based TCT optimization framework specifically designed for linear repetitive construction projects, addressing a critical gap in current scheduling methodologies. By integrating GA and PSO with LOB scheduling, the proposed framework significantly enhances decision-making in construction project management. This enhancement is achieved through systematic task decomposition and dynamic crew allocation, which enable more effective resource management. A comparative evaluation of GA and PSO within a consistent problem setting revealed notable improvements in cost efficiency and project duration, underscoring the practical applicability of metaheuristic optimization in repetitive construction environments.

In terms of cost performance, key findings highlight the superior efficiency of the PSO model for linear repetitive construction projects. Specifically, the PSO approach achieved a direct cost reduction of 4%, decreasing from 45,617,920 EGP to 43,791,640 EGP for four units. Additionally, it accomplished a significant 20% reduction in indirect costs, falling from 8,316,000 EGP to 6,648,000 EGP. Overall, the total construction cost was reduced by 10.9%, decreasing from 56,621,920 EGP to 50,439,640 EGP. In contrast, the GA model also demonstrated effective cost management, realizing a 3.25% decrease in direct costs, bringing them down to 44,135,520 EGP. It matched the PSO’s 20% reduction in indirect costs, resulting in a total indirect cost of 6,648,000 EGP. Consequently, the total construction cost for the GA model was reduced by 7%, amounting to 52,835,520 EGP. Regarding time performance, both the PSO and GA models revealed substantial improvements in project timelines. For four-unit projects, the proposed approaches reduced project duration from 693 days to 554 days, representing a notable 20% improvement in time efficiency.

These results illuminate the advantages of metaheuristic optimization over traditional scheduling techniques, particularly in managing the complexities of linear repetitive projects. The decomposition of repetitive tasks into smaller sub-tasks allows for more granular control over scheduling constraints, leading to optimized resource utilization and minimized idle time. The comparative analysis between GA and PSO revealed that while both algorithms effectively optimized project schedules, PSO exhibited slightly better cost efficiency, whereas GA provided robust exploration of diverse solution spaces. This distinction suggests that PSO may be preferable for rapid convergence in time-sensitive projects, while GA could be advantageous in scenarios requiring extensive solution space exploration. The integration of LOB with metaheuristic optimization further enhanced scheduling flexibility, ensuring continuous workflow and efficient crew allocation, which is a critical factor in large-scale repetitive construction.

This research contributes to both theoretical and practical advancements in construction project management. Theoretically, it bridges the gap between traditional LOB scheduling and advanced optimization techniques, offering a structured methodology for future studies in repetitive project optimization. Practically, the framework provides project managers with a scalable decision-support tool that improves cost estimation, reduces delays, and enhances operational efficiency. Future research could explore hybrid metaheuristic models, sensitivity analysis of optimization parameters, and real-world implementation across diverse construction scenarios to further validate the framework’s robustness.

In conclusion, this study establishes a novel, replicable approach for optimizing time-cost trade-offs in linear repetitive projects, demonstrating that metaheuristic algorithms, when integrated with LOB scheduling, can significantly outperform conventional methods. By advancing the integration of optimization techniques with construction scheduling, this research paves the way for more efficient, cost-effective, and adaptive project management practices in the construction industry.

No potential conflict of interest was reported by the authors.

## Data Availability

This manuscript does not report data generation or analysis. Ahmed Gouda Mohamed will be responsible if someone wants to request the data from this study.

## Abbreviations

TCT: Time-cost trade-off
TCQT: Time-cost-quality trade-off
TCET: Time-cost-environmental impact trade-off
TCTP: Time-cost trade-off problem
LRPs: Linear repetitive projects
LRPTCT: Linear repetitive project time-cost trade-off
NRP: Non-unit repetitive projects
LOB: Line of balance
CPM: Critical path method
GA: Genetic algorithm
PSO: Particle swarm optimization
ACO: Ant colony optimization
GROL: Golden ratio-based oppositional learning
MDOLTLBO: Modified dynamic opposition learning-assisted teaching-learning-based optimization
MOWO: Multiple objective whale optimization
NSGA-II: Nondominated sorting genetic algorithm-II
TLBO: Teaching-learning-based optimization
DTCTP: Discrete time-cost trade-off problem
MILP: Mixed-integer linear programming
NDS: Non-dominant sorting
TDC: Total direct costs
TIC: Total indirect costs
TCC: Total construction cost
ω: Inertia weight
c1: Cognitive coefficient
c2: Social coefficient
r1 and r2: Random factors
p_best: Best-known positions
g_best: Global best position
Rd: Desired delivery rate for the repetitive units
n: Number of repetitive units
TL: Project’s deadline duration
T1: CPM duration of the first unit
Tfi: Total float of each sub-task (i)
Ci: Number of crews assigned to each sub-task (i)
Ri: Desired delivery rate for each sub-task (i)
Di: Duration of sub-task (i)
